# Biophysical and Computational Insights into Alpha-1 Antitrypsin Aggregation and Its Inhibition by Natural Polyphenols

**DOI:** 10.3390/biomedicines14061310

**Published:** 2026-06-09

**Authors:** Tarique Sarwar, Ahmed Abdur Rehman, Hussain Arif, Wanian M. Alwanian, Hajed Obaid A. Alharbi, Arshad Husain Rahmani

**Affiliations:** 1 Department of Medical Laboratories, College of Applied Medical Sciences, Qassim University, Buraydah 51452, Saudi Arabia; t.sarwar@qu.edu.sa (T.S.); w.alwanian@qu.edu.sa (W.M.A.); hajed.alharbi@qu.edu.sa (H.O.A.A.); 2 Department of Biochemistry, Faculty of Life Sciences, Aligarh Muslim University, Aligarh 202002, India; ahmedarehman@gmail.com (A.A.R.); arifkap@gmail.com (H.A.)

**Keywords:** alpha-1-antitrypsin, protein, amentoflavone, theaflavin, aggregation, molecular dynamics simulation, polyphenols, amyloid fibrils, molten globule, inhibition

## Abstract

**Background/Objectives**: Protein misfolding and amyloid fibril formation underlie several degenerative diseases, including Alzheimer’s disease and Parkinson’s disease. Alpha-1 antitrypsin (A1AT), a serpin protein, is particularly prone to misfolding, with polymerization and aggregation implicated in alpha-1 antitrypsin deficiency and associated hepatic and pulmonary disorders. In this study, we examined the structural changes in A1AT induced by the fluorinated alcohol, trifluoroethanol (TFE), and assessed the inhibitory effects of two natural polyphenols, amentoflavone (AMF) and theaflavin (TF), on aggregation and fibril formation. **Methods**: A library of selected phytocompounds was virtually screened against the crystal structure of A1AT (PDB 3NE4) using AutoDock Vina to elucidate their binding affinity towards it. Based on binding affinities, two compounds, AMF and TF, were selected for further studies. Protein aggregation was induced with TFE, and the protective effects of AMF and TF were evaluated using protease inhibitory activity, intrinsic fluorescence, turbidity, Rayleigh scattering, ANS fluorescence, and ThT fluorescence assays. Furthermore, 100 ns molecular dynamics simulation and MM-PBSA calculations were performed to assess the stability and binding interactions of the A1AT–ligand complexes. **Results**: Pre-treatment of A1AT with AMF or TF significantly inhibited TFE-induced aggregation in a dose-dependent manner, with AMF being consistently more effective. ThT fluorescence analysis revealed a ~60–65% decrease in aggregate formation upon treatment with polyphenols, with IC_50_ values estimated at ~40 µM for AMF and ~50 µM for TF, both of which are statistically significant. Molecular docking and 100 ns molecular dynamics simulation also revealed stable A1AT–polyphenol interactions, with AMF exhibiting greater binding affinity and greater attenuation of solvent-induced conformational perturbation. **Conclusions**: Collectively, our findings show that TFE causes A1AT misfolding via a molten globule-like intermediate, resulting in fibril formation at 30–40% TFE, and natural polyphenols AMF and TF inhibited aggregation in a concentration-dependent manner. These observations suggest the potential of AMF and TF as lead scaffolds for anti-aggregation strategies, as modulators of amyloidogenic processes.

## 1. Introduction

Protein and peptide folding is critical to proper biological function. Early, misfolded proteins are present as monomers with varying degrees of folding, forming disordered, partially folded, or near-native oligomers. Chronic amyloid structures form more stable aggregates, characterized by dense, larger units with high β-sheet content. These aggregates can further aggregate into fibrils with a cross-β structure, which can accumulate in body tissues [1]. This abnormal protein aggregation forms the basis of various amyloid-related diseases, such as Alzheimer’s disease, type II diabetes, Parkinson’s disease, familial amyloidosis, and Huntington’s disease [2].

Alpha-1 antitrypsin (A1AT) is a plasma glycoprotein with a molecular mass of about 52 kDa, best known as an extracellular inhibitor of neutrophil elastase (NE). It is the major circulating serine protease inhibitor, encoded by the SERPINA1 gene on chromosome 14q32.1–32 [3]. The serine protease inhibitors (SERPINs) belong to a family with identical gene sequences and structural motifs [4,5]. A1AT is one of the most common antiproteases in the blood, present at approximately 1.3 g/L, with a physiological range of 0.9 to 1.75 g/L, and a plasma half-life of approximately 4 to 5 days. Although hepatocytes are the major source of circulating A1AT, other cells, such as monocytes, macrophages, alveolar epithelial cells, and intestinal epithelial cells, also produce smaller amounts of A1AT [6,7,8]. During inflammation, as part of the acute-phase reaction, its plasma levels can rise two- to fourfold, largely in response to pro-inflammatory cytokines such as interleukin-6 (IL-6), interleukin-1β (IL-1β), and, to a lesser extent, IL-8, transforming growth factor-β (TGF-β), and IL-17 [9]. Its rise in systemic and local A1AT concentrations increases its ability to regulate inflammation by binding proteases, peptides, and proteins and by interacting with cell-surface receptors [10,11].

Conversely, low serum A1AT levels lead to uncontrolled neutrophil elastase activity, which contributes to the onset of numerous diseases, chiefly emphysema [12]. Structurally, upon transcription and translation, A1AT assumes a tertiary conformation consisting of three β-sheets, nine α-helices, and a reactive center loop (RCL) towards the C-terminus [13,14]. Upon release into the blood, about 80% of circulating A1AT diffuses into interstitial tissues, and a smaller fraction (0.5–10%) is localized in other body fluids, including alveolar fluid, where it exists in concentrations between 0.1 and 0.3 g/L [15].

Research using X-ray crystallography, computational modeling, and kinetic analysis has contributed toward understanding the folding kinetics of A1AT [16,17]. Serpins’ conformational flexibility is requisite for their inhibitory activity, but it also makes them susceptible to misfolding and aggregation. During folding, A1AT assumes a metastable state in which a 15-amino acid segment (residues 345–360) in its C-terminal region, the RCL, is maintained in an open conformation, exposed to the surrounding aqueous solvent. This loop, which links the β-s5A and β-s1C strands, is mobile and primarily responsible for protease targeting. On binding with a protease, there is a profound structural change in A1AT, caused by cleavage at the P1′–P1 bond within the RCL, triggering the mode of inhibition [18].

Amentoflavone (AMF) is a naturally occurring plant biflavonoid present in a broad range of plant species. It was first isolated in 1971 by Okigawa et al. from three species of Selaginella, including *Selaginella tamariscina* (Beauv.) Spring, *Selaginella nipponica*, and *Selaginella pachystachys* [19]. Pharmacological studies have identified many biological activities of AMF, including antioxidant [20], anti-inflammatory [21], anti-aging [22], anticancer [23], antiviral [24], and antifungal [25] activities. It has also shown therapeutic usefulness in disorders of the central nervous system [26] and the cardiovascular system [27]. Due to its notable pharmacological advantages and high natural occurrence, AMF has been identified as a chemical marker for quality control of Selaginellae Herba (“Juanbai” in Chinese), including the entire plants of *Selaginella tamariscina* and *Selaginella pulvinata*, according to the Chinese Pharmacopeia [28]. The pluralistic biological activities and natural sources of AMF have rendered them a growing focus of scientific research across various fields.

Theaflavins (TF) are major pigments in black tea and contribute to its characteristic color and flavor [29]. It is a product of catechin oxidation during black tea processing and is one of the major contributors to the beneficial effects observed with black tea consumption [30]. This bioactive pigment has shown a variety of beneficial effects, including antioxidant [31], anticancer [32], anti-inflammatory [33], antibacterial [34], blood-sugar-lowering [35], and lipid-lowering [36] activities. It has recently been demonstrated that TF prevents oxidative stress by activating the Nrf2 signaling pathway, thereby alleviating brain ischemia/reperfusion (I/R) injury [37] and protecting hematopoietic stem cells from ionizing radiation-induced damage [38].

AMF and TF were selected due to their reported anti-amyloid, antioxidant, and protein-stabilizing properties, as well as their ability to modulate aggregation pathways in several amyloidogenic proteins. These features make them promising candidates to evaluate as potential inhibitors of A1AT aggregation.

Investigating A1AT accumulation is significant, as it is a driving force in the pathophysiology of alpha-1-antitrypsin deficiency (A1ATD), an inherited disease that may cause fatal lung and liver disease. Some mutations, such as the Z allele (Glu342Lys), result in misfolded proteins that aggregate into polymers in hepatocytes (liver cells) [39]. The accumulation may result in liver injury, including hepatitis, cirrhosis, and, eventually, hepatocellular carcinoma. The understanding of aggregation sheds light on why 10–15% of adults with the ZZ genotype acquire liver disease and why 3–5% of children with the ZZ mutation are confronted with life-threatening liver illness [40]. Though aggregation is largely responsible for liver injury, the misfolded A1AT proteins do not leave the cell, resulting in low blood and lung levels of functional A1AT. This deficit exposes the lungs to neutrophil elastase, an elastolytic enzyme, leading to conditions such as emphysema and COPD [41]. Investigating aggregation helps explain how the absence of active A1AT underlies such lung conditions, particularly in ZZ or SZ genotypes. Research on A1AT aggregation provides opportunities to develop therapies that inhibit polymerization and may decrease liver damage.

Several approaches have been explored to inhibit A1AT aggregation, including small molecules, peptides, and pharmacological chaperones that stabilize native conformations or prevent polymerization. However, many of these compounds suffer from limited bioavailability, toxicity, or lack of specificity. Moreover, despite their well-known anti-amyloidogenic effects in other protein systems, naturally occurring polyphenols remain underexplored in the context of A1AT aggregation. This highlights the need to identify safe, naturally derived modulators that can interfere with A1AT misfolding pathways.

Despite extensive studies on genetic A1AT polymerization, limited work has explored chemically induced aggregation pathways and their modulation by natural small molecules under controlled conditions [42,43]. Organic solvent-induced misfolding, particularly with fluorinated alcohols, is a well-established approach for generating aggregation-prone intermediates and amyloid-like fibrils in vitro. Trifluoroethanol (TFE) is widely used to mimic membrane-like environments and to stabilize partially folded intermediates that promote amyloid formation. Unlike chaotropic denaturants such as urea or guanidinium chloride, which cause complete unfolding, TFE preferentially stabilizes secondary structural elements while destabilizing tertiary contacts, thereby favoring aggregation-prone conformations. Studying chemically induced aggregation provides a controlled, reproducible platform for screening aggregation inhibitors. This study uses TFE-induced misfolding of A1AT as a model to examine the anti-aggregation effects of the natural polyphenols AMF and TF, which are known modulators of amyloidogenesis in other proteins. We assess whether TFE-induced misfolding involves a molten globule-like intermediate and if AMF and TF can influence this process. Using biophysical assays and MD simulations, we aim to define A1AT states, evaluate the effects of AMF and TF on aggregation and ThT-positive species, and analyze the stability of A1AT–polyphenol complexes.

## 2. Materials

AMF (>98% purity) and TF (>98% purity) were purchased from Sigma Aldrich (St. Louis, MO, USA). A1AT (>95% purity) was purchased from Abcam, Cambridge, UK. Sodium phosphate monobasic and sodium phosphate dibasic were purchased from Merck, USA. Trifluoroethanol (≥99.5% purity), 8-anilino1-naphthalene sulphonic acid (ANS), and thioflavin-T (ThT) were obtained from Sigma Aldrich (St. Louis, MO, USA). Sodium phosphate mono and dibasic (pH 7.4) were used for buffer preparations. Sodium phosphate buffers (50 mM, pH 7.4) were prepared from sodium phosphate mono- and dibasic salts using Milli-Q water and filtered prior to use.

## 3. Methods

### 3.1. Virtual Screening of Phytocompounds

Molecular docking is an effective computational technique used in structure-based drug design, enabling the prediction of ligand-binding orientation and affinity. In this study, AutoDock Vina was employed to perform docking simulations against A1AT to explore potential binding interactions with selected phytocompounds [44,45]. The A1AT crystal structure (PDB ID: 3NE4), obtained from the RCSB Protein Data Bank (https://www.rcsb.org, accessed on 5 October 2025), was prepared by removing non-protein heteroatoms and water molecules. Preparation steps also included adding Kollman charges and merging non-polar hydrogens using AutoDock Tools (v1.5.6), followed by conversion to PDBQT format. A docking grid was configured with a spacing of 1.0 Å and dimensions of 74 × 50 × 60 Å, centered at x = 14.447, y = −0.002, z = 22.901, covering the functional binding pocket. Phytochemical ligands were sourced on 5 October 2025, from the PubChem database (https://pubchem.ncbi.nlm.nih.gov/) in SDF format and converted to PDB files with UCSF Chimera (v1.14). Ligand preparation involved processing PDB files using AutoDock Tools and identifying RMSD minima to ensure ligand flexibility and suitable conformations. This involved assigning torsion trees and defining flexible bonds to facilitate efficient conformational sampling during docking. Final ligand structures were saved in PDBQT format. After docking, the binding modes and interaction poses were examined using Discovery Studio Visualizer 2021 and PyMOL (v3.1.3) to visualize hydrogen bonds, hydrophobic contacts, and the spatial fit within the receptor site [44]. To provide internal validation of the docking protocol, we verified that repeated docking runs for representative ligands yielded consistent poses that occupied the same pocket and engaged similar residues, indicating robust sampling of the intended site. AMF and TF were selected for further study on the basis of their predicted binding energies, favorable interaction networks, and previously reported anti-amyloidogenic and protein-stabilizing properties. This comprehensive docking approach identified candidate phytochemicals with potential affinity for A1AT for further in silico and experimental validation.

### 3.2. Preparation of Samples

A1AT was prepared at a concentration of 0.5 mg/mL in 50 mM sodium phosphate buffer (pH 7.4) and incubated with trifluoroethanol (TFE) at concentrations ranging from 0% to 70% (*v*/*v*). Incubation was at 37 °C for 2 h to allow equilibration prior to spectroscopic examination. AMF and TF were initially dissolved in DMSO to prepare 10 mM stock solutions, which were then diluted in phosphate buffer to achieve a final DMSO concentration of 0.5–1% (*v*/*v*). These solutions showed no measurable effect on A1AT structure or activity. Control experiments confirmed that equivalent concentrations of DMSO alone did not affect protein aggregation or fluorescence. For spectroscopic assays, the final A1AT concentration was adjusted to 10 µM unless otherwise indicated. All experimental conditions were maintained unless otherwise noted, and each assay was repeated 3 times for reproducibility. TFE was chosen as our model denaturant because it stabilizes partially folded intermediates and molten globule-like states without full unfolding. The TFE concentrations were determined from transition points that reflect protein dynamics. Lower concentrations promoted intermediate states, whereas higher concentrations favored aggregation-prone conformations, underscoring TFE’s vital role in protein stability.

### 3.3. Assay of A1AT Activity

A1AT protease inhibitory activity was assessed by its ability to inhibit trypsin-mediated cleavage of the chromogenic substrate Nα-Benzoyl-DL-arginine 4-nitroanilide hydrochloride (BAPNA), as described previously [46]. After incubating A1AT with the desired TFE concentration (0–70% *v*/*v*) in the presence or absence of AMF/TF for 2 h at 37 °C, samples were equilibrated to room temperature and then mixed with trypsin and BAPNA in 50 mM sodium phosphate buffer (pH 7.4). Reaction progress was monitored at 405 nm using a UV–Visible spectrophotometer (UV-1700, Shimadzu, Tokyo, Japan). Native A1AT without TFE served as the 100% activity control. Relative inhibitory activity was calculated from the slope of absorbance vs. time relative to the control. The TFE titration (0–70% *v*/*v*) was first performed without inhibitors to define the solvent dependence of A1AT activity; the 40% TFE condition, at which maximal loss of activity was observed, was then used as the stress condition to assess the ability of AMF and TF to preserve activity.

### 3.4. Intrinsic Fluorescence Measurements

Fluorescence emission spectra were obtained using a Shimadzu RF-5301PC spectrofluorometer (Tokyo, Japan) with a 1-cm path-length quartz cuvette. Protein samples were excited at 280 nm to selectively excite aromatic residues, primarily tryptophan, and the emission spectra were scanned between 300 and 400 nm [47]. Both excitation and emission slit widths were kept at 5 nm. The final concentration of A1AT in each sample was 10 μM, whereas the test phytocompounds, AMF and TF, were screened at concentrations of 10–100 μM to assess concentration-dependent interactions. For each TFE concentration, spectra were acquired for (i) buffer with TFE and DMSO only, (ii) buffer with TFE and DMSO plus AMF or TF, and (iii) full samples containing A1AT with/without AMF or TF. Spectra from buffer and compound blanks were subtracted from the corresponding protein-containing spectra to remove background fluorescence and account for TFE- and DMSO-dependent changes in refractive index and inner filter effects.

### 3.5. Turbidity Assay

Turbidity measurements were used as a qualitative readout of insoluble aggregate formation [48]. A1AT samples (10 µM) were incubated with 0–70% (*v*/*v*) TFE at 37 °C for 2 h, and absorbance was measured at 350 nm using a UV–Visible spectrophotometer with a 1 cm path-length quartz cuvette. A buffer containing equal amounts of TFE and DMSO, but without protein, was used as a blank. To assess the effects of AMF and TF, A1AT was pre-incubated with increasing concentrations of the polyphenols (10–100 µM) for 30 min at 37 °C, then 40% TFE (*v*/*v*) was added, and the mixture was incubated for 2 h. The 40% TFE condition was selected because it produced the highest turbidity in the titration experiments, corresponding to maximal aggregate burden. Turbidity values for inhibitor-treated samples were expressed relative to the A1AT + 40% TFE control.

### 3.6. Rayleigh Scattering Measurements

Rayleigh scattering measurements were performed to analyze variations in light-scattering characteristics that reflect protein aggregation or complex formation. The experiments were performed using a Shimadzu RF-5301PC spectrofluorometer (Tokyo, Japan) equipped with a 1-cm path-length quartz cuvette. The excitation wavelength was set to 350 nm, and the emission spectra were recorded over the 300–400 nm range. Both excitation and emission slit widths were kept constant at 5 nm. Buffer and solvent blanks (with TFE and DMSO, with or without AMF/TF) were recorded and subtracted to isolate scattering from protein-containing samples. Integrated scattering intensity over the emission range was used as a measure of aggregate formation. For inhibition experiments, 40% TFE (*v*/*v*) was again used as the stress condition, corresponding to the maximum scattering observed in the titration. A1AT was pre-incubated with AMF or TF (10–100 µM) for 30 min at 37 °C prior to the addition of TFE and a further 2 h incubation.

### 3.7. ANS Fluorescence Measurements

8-Anilinonaphthalene-1-sulfonic acid (ANS), a hydrophobic fluorescent probe, was used to quantify conformational changes and surface hydrophobicity in A1AT [49]. Emission fluorescence spectra were measured with excitation at 380 nm, and emission scanned from 400 to 600 nm to detect ANS-specific fluorescence. The ANS concentration was kept at a 100-fold molar excess over the protein to achieve saturation binding and a maximum fluorescence response. A1AT’s final concentration was 10 μM, whereas the phytocompounds, AMF and TF, were examined between concentrations of 10 and 100 μM.

### 3.8. ThT Fluorescence Assay

The ThT fluorescence assay was used to detect amyloid-like fibril formation in A1AT under diverse treatment conditions. The assay exploits the amplified fluorescence of ThT upon binding to β-sheet-rich amyloid assemblies [50]. Fluorescence was measured using a 1 cm path-length quartz cuvette, excited at 440 nm, and emission spectra were recorded from 450 to 600 nm. ThT was dissolved in 50 mM sodium phosphate buffer (pH 7.4). The final concentration of A1AT was 10 μM, whereas the concentration of ThT was 40 μM. The phytocompounds, AMF, and TF were added at concentrations ranging from 10 to 100 μM to assess their potential anti-amyloidogenic activity. For inhibitor studies, A1AT was pre-incubated with AMF or TF (10–100 µM) for 30 min at 37 °C, then 40% TFE was added and incubated for 2 h before ThT addition and fluorescence measurement. ThT data were collected as single-endpoint measurements after 2 h incubation; no time-resolved kinetic curves were acquired in this study. Consequently, our ThT analysis reports steady-state aggregate burden rather than nucleation or elongation rate constants.

### 3.9. Molecular Dynamics Simulation

To further understand the dynamic stability and interaction profiles of the best-performing phytocompounds, AMF, and TF against A1AT, molecular dynamics (MD) simulations were conducted. The best docking conformations with the lowest binding energy were taken as the initial structures for the simulations. The MD simulations were performed using the GROMACS 2018.1 package with the amber99sb-ILDN force field applied to the protein [51,52]. Ligand topologies were prepared using the Antechamber module of AmberTools21 to ensure compatibility with the chosen force field [53]. As a control, an unbound A1AT simulation was also run under the same conditions. Each system was embedded in a triclinic simulation box and solvated through the TIP3P explicit water model. To approximate physiological conditions, three chloride anions were added to counterbalance the system, and 150 mM NaCl was added to mimic ionic strength. Energy minimization was performed before production runs using the steepest-descent algorithm for up to 50,000 steps to remove unfavorable steric interactions and stabilize the initial structures.

The equilibration process consisted of a 1 ns simulation at constant volume and temperature (NVT) at 310 K with the V-rescale thermostat, followed by a 1 ns constant pressure (NPT) equilibration at 1 bar with the Parrinello–Rahman barostat [54,55]. After that, a 100 ns production run was performed for all systems under periodic boundary conditions. Simulation trajectories were all corrected for the corresponding periodic boundary condition (PBC) before analysis. Important structural and dynamic properties, such as backbone root mean square deviation (RMSD), root mean square fluctuation (RMSF), solvent-accessible surface area (SASA), and radius of gyration (RG), were calculated to evaluate protein stability and conformational freedom. Furthermore, hydrogen-bonding analysis and secondary structure evaluations were performed to gain insight into the structural integrity of A1AT upon complexation with the ligand. Principal component analysis (PCA) was performed using the gmx covar and gmx anaeig utilities within GROMACS to analyze dominant motions and conformational sampling throughout the simulation. Additionally, binding free energies for the A1AT-ligand complexes were estimated using the Molecular Mechanics Poisson–Boltzmann Surface Area (MM-PBSA) approach, which delivers thermodynamic information related to the affinity and stability of the interactions [56].

### 3.10. Statistical Analysis

All experiments had at least three replicates, with data reported as mean ± SD unless noted otherwise. Biophysical assays were analyzed using one-way ANOVA and Tukey’s test for multiple comparisons. *p*-values (* *p* < 0.05, ** *p* < 0.01). For concentration-dependent inhibition of ThT-positive aggregates by AMF and TF at 40% TFE, fluorescence was modeled to determine IC50, with intervals and fit metrics reported in the main text or in the legends.

## 4. Results

### 4.1. Virtual Screening of Phytocompounds

**Figure 1:**
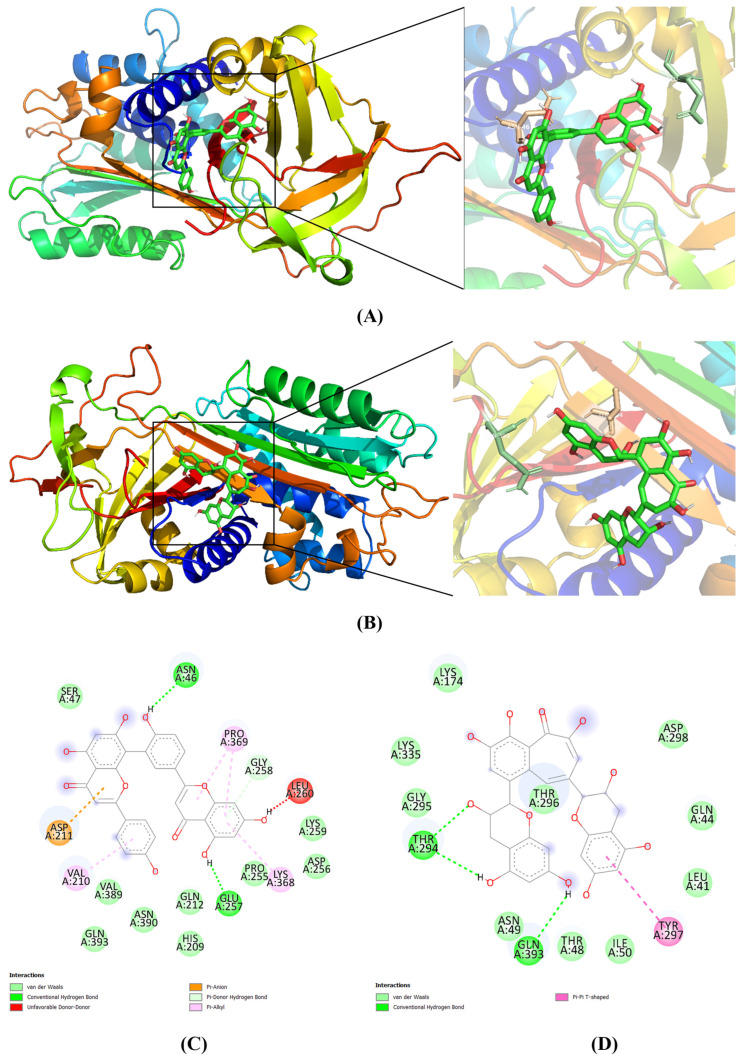
Molecular docking showing a (**A**) 3D model with detailed view of the docking pose of A1AT complexed with (**A**) AMF and (**B**) TF. A 2D plot of the interaction of A1AT with (**C**) AMF and (**D**) TF.

Molecular docking, a crucial step in virtual screening, was performed to assess interactions between phytocompounds and A1AT. Docking was conducted with the AutoDock Vina, and the binding energies were calculated and tabulated in Table S1 (Supplementary Information). Good affinities were observed for most compounds, with binding energies ranging from −5.4 to −8.6 kcal/mol. Of these, AMF and TF were identified as the leading contenders based on binding-site complementarity, binding energies, pharmacokinetic properties, ADMET features, and predicted anti-fibrillar and anti-amyloidogenic activities. Both compounds fit well into the binding pocket, stabilized by diverse non-covalent interactions (Figure 1A,B), which underpin their high affinities. Structural interaction analysis showed that AMF formed a hydrogen bond with Asn46 and Glu257, a π-anion interaction with Asp211, a π-donor hydrogen bond with Gly258, and multiple van der Waals contacts, as well as an unfavorable donor–donor contact with Leu260 (Figure 1C). TF formed hydrogen bonds with Thr294 and Gln393, a π–π T-shaped interaction with Tyr297, as well as multiple van der Waals contacts (Figure 1D). TF forms more hydrogen bonds with the protein, suggesting a stronger enthalpic contribution to binding. However, AMF exhibited a stronger overall binding affinity, likely due to enhanced hydrophobic interactions and van der Waals interactions that collectively stabilize the complex more effectively. These contacts suggest that AMF and TF binding induce minor conformational changes in A1AT, thereby contributing to their expected anti-amyloidogenic action. Overall, these findings place AMF and TF as valuable contenders for validation through further in vitro and in vivo testing.

### 4.2. A1AT Activity

**Figure 2:**
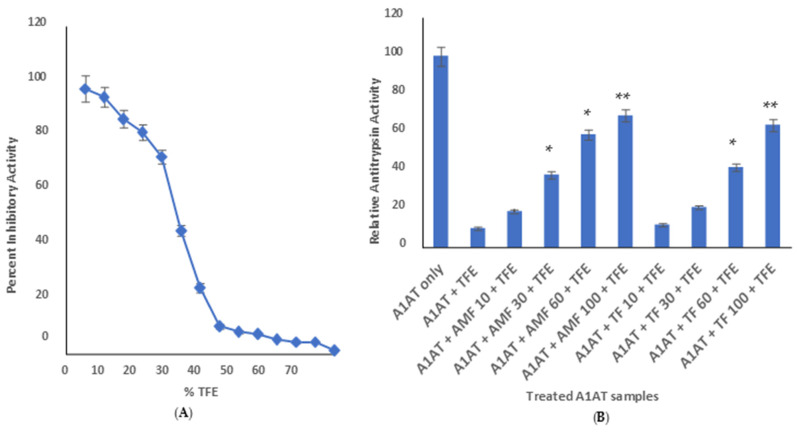
(**A**) Effect of increasing concentration of TFE on inhibitory activity of A1AT: A1AT was incubated with increasing concentrations (10–70% *v*/*v*) of TFE for 2 h at room temperature and then assayed for antiproteolytic activity. (**B**) Effect of pre-treatment of A1AT with increasing concentrations of polyphenols AMF and TF, on the protease inhibitory activity. Four different concentrations (10, 30, 60, 100 µM) of each AMF and TF were used in the study. The TFE concentration used as a control is 40% (*v*/*v*). Statistical significance was assessed using one-way ANOVA followed by Tukey’s multiple comparisons test. Comparisons are shown relative to the A1AT + TFE group unless otherwise indicated (* *p* < 0.05, ** *p* < 0.01).

Prior to examining the biophysical behavior of A1AT, the effect of the organic solvent trifluoroethanol (TFE) on its protease inhibition activity was characterized (Figure 2A). In the absence of solvent, native A1AT showed the highest antitrypsin activity and served as the control. When the TFE concentration was progressively raised, there was a negligible alteration in inhibitory activity up to 10% (*v*/*v*). Nevertheless, above this level, activity fell sharply, reaching a minimum of 40% (*v*/*v*) TFE. Such solvent-induced loss of activity is generally attributed to reduced accessible surface area and conformational flexibility of proteins. In aqueous solutions, proteins possess conformational mobility necessary for their biological activity, while organic solvents limit this flexibility [57]. With increased TFE concentration, the reduced solvent capacity to form multiple hydrogen bonds promotes stronger intramolecular electrostatic forces, leading to a more rigid protein structure and a loss of activity [58]. This indicates that A1AT retains its inhibitory activity under modest solvent stress but loses activity when challenged with high levels of TFE.

Interestingly, pre-incubation with increasing concentrations of the polyphenols AMF and TF substantially mitigated the loss of inhibitory activity induced by TFE (Figure 2B). Activity restoration was dose-dependent, which means that both polyphenols stabilized the protein and maintained its functional conformation under solvent stress. Between the two, AMF demonstrated greater protective action than TF, reflecting its better capacity to reduce TFE-induced functional inactivation of A1AT. Although high TFE concentrations alter solvent polarity, control experiments confirmed that AMF and TF retain measurable inhibitory effects even at elevated TFE levels, indicating that their interaction with A1AT is not abolished under these conditions. However, we acknowledge that high TFE may modulate compound–protein interactions, which is a limitation of solvent-induced aggregation models.

### 4.3. Intrinsic Fluorescence

**Figure 3:**
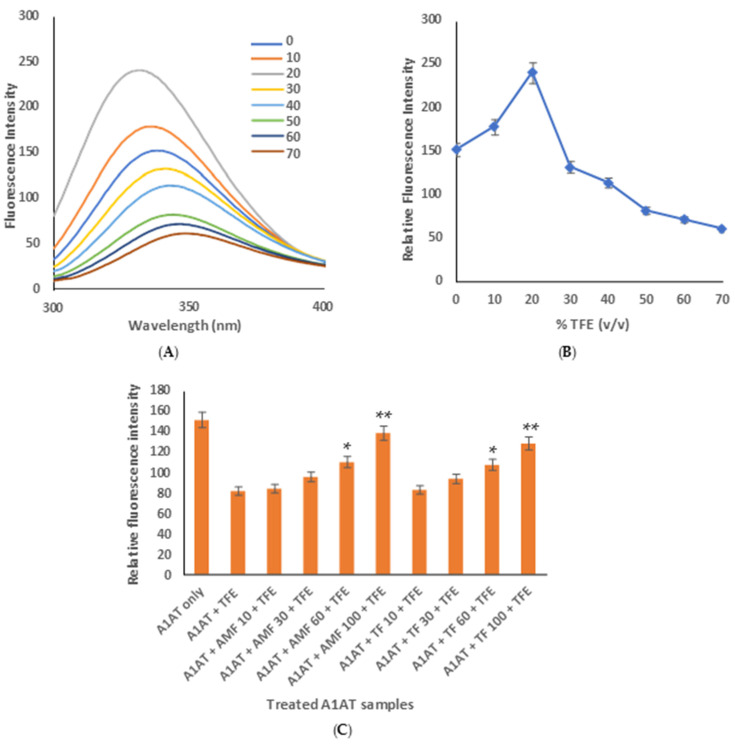
Intrinsic fluorescence measurement of native and treated A1AT: (**A**) Intrinsic fluorescence emission spectra of native and TFE (10–70% *v*/*v*) treated A1AT. (**B**) Relative intrinsic fluorescence pattern of native and TFE-treated A1AT. Peaks achieved on the respective TFE concentrations were plotted. (**C**) The effect of increasing concentrations of polyphenols, AMF, and TF on TFE-induced structural and conformational alterations in A1AT as monitored by intrinsic fluorescence. Four different concentrations (10, 30, 60, and 100 µM) of both AMF and TF were used for the study. The TFE concentration used as a control is 50% (*v*/*v*). Statistical significance was assessed using one-way ANOVA followed by Tukey’s multiple comparisons test. Comparisons are shown relative to the A1AT + TFE group unless otherwise indicated (* *p* < 0.05, ** *p* < 0.01).

Intrinsic fluorescence spectroscopy is a commonly used method for monitoring protein conformational changes by sensing the microenvironment of aromatic residues, especially tryptophans [59]. TFE-induced change in the fluorescence properties of A1AT is depicted in Figure 3. As illustrated in Figure 3B, the relative fluorescence intensity initially increased, peaking at 20% (*v*/*v*) TFE, then dropped sharply to 30%, and gradually declined, eventually leveling off beyond 50%. The emission spectra shown in Figure 3A revealed that native A1AT exhibited an emission maximum near 338 nm. Upon treatment with 10% TFE, fluorescence intensity increased along with a 2 nm blue shift; at 20% TFE, the intensity increased further, accompanied by a significant 7 nm blue shift relative to the native protein. These blue shifts suggest that tryptophan residues are moving into a more hydrophobic environment, signaling a structural rearrangement within the protein core. Such changes indicate the formation of a partially folded intermediate, likely a molten globule-like conformation of A1AT at these solvent concentrations [60].

At higher TFE concentrations (30–40%), a significant reduction in fluorescence intensity and red shifts of ~2 nm and ~5 nm, respectively, were observed. An additional increase in TFE (50–70%) led to a progressive decrease in intensity, followed by further red shifts. The spectral trend in this case indicates exposure of tryptophan residues to the solvent, thereby disrupting the tertiary structure. TFE at higher concentrations has been reported to weaken hydrophobic interactions and strengthen solvent–protein polar interactions [61], thus destabilizing the native fold. The observed data therefore show that moderate TFE levels promote a dense, molten globule-like intermediate, whereas elevated concentrations cause unfolding and full exposure of Trp residues to the water phase, indicative of loss of tertiary structure [62].

The effect of polyphenols on the intrinsic fluorescence of TFE-treated A1AT is represented in Figure 3C. Pre-incubation with progressively higher concentrations of AMF and TF recovered fluorescence intensity in a dose-dependent manner toward that of the native protein, indicating that these compounds resisted solvent-induced conformational changes. Interestingly, AMF had a greater protective effect than TF as it was more effective in maintaining native-like spectral characteristics. These results imply that polyphenols, especially AMF, stabilize A1AT by preventing solvent-induced over-unfolding and may also function as molecular shields against structural perturbation. Since TFE itself influences fluorescence properties by altering solvent polarity and refractive index, control experiments were performed in which fluorescence changes due to TFE alone were monitored and subtracted or compared against protein-free TFE controls.

### 4.4. ANS Fluorescence Measurements

To probe intermediate states along the A1AT unfolding pathway from the folded to the unfolded conformation, 8-anilino-1-naphthalene sulfonic acid (ANS), a hydrophobic fluorescent probe, was used. ANS can bind noncovalently to exposed hydrophobic patches that are normally buried in the native protein interior [63]. A rise in ANS fluorescence relative to the native state thus indicates unfolding or structural rearrangements that expose hydrophobic residues to the solvent [64].

**Figure 4:**
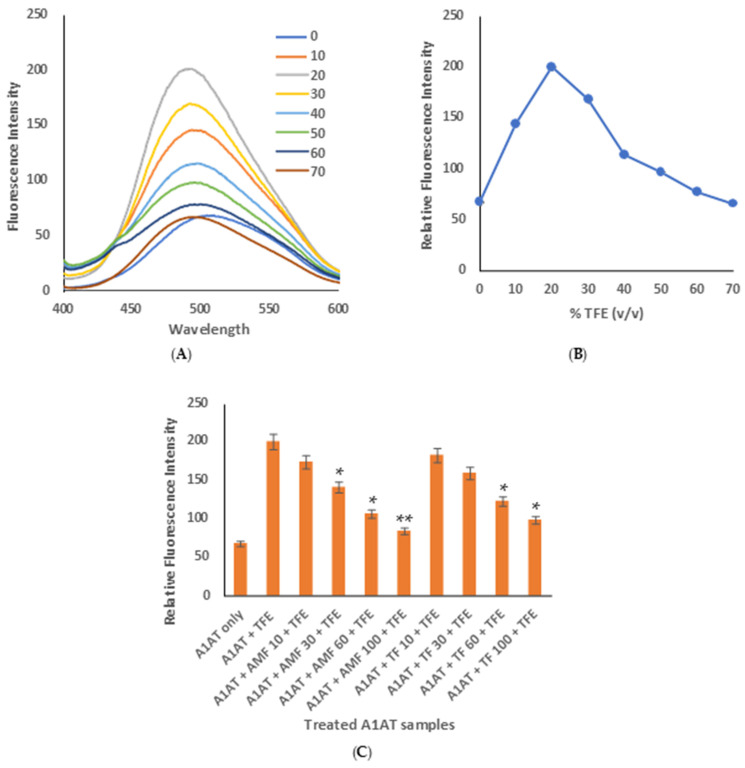
ANS fluorescence measurement of native and treated A1AT: (**A**) ANS fluorescence spectra of native and TFE (10–70% *v*/*v*) treated A1AT. (**B**) Relative fluorescence pattern of ANS bound to native and TFE-treated A1AT. Peaks achieved on the respective TFE concentrations were plotted. (**C**) The effect of increasing concentrations of polyphenols, AMF, and TF on TFE-induced structural and conformational alterations in A1AT, as monitored by ANS fluorescence. Four different concentrations (10, 30, 60, and 100 µM) of both AMF and TF were used for the study. The TFE concentration used as a control is 20% (*v*/*v*). Statistical significance was assessed using one-way ANOVA followed by Tukey’s multiple comparisons test. Comparisons are shown relative to the A1AT + TFE group unless otherwise indicated (* *p* < 0.05, ** *p* < 0.01).

As indicated in Figure 4A, the ANS fluorescence spectrum of native A1AT was very weak with an emission maximum at about 504 nm. The faint signal indicates minimal exposure of hydrophobic residues, consistent with a compact, native tertiary structure in which such residues are considerably buried in the core [65]. In contrast, treatment with 10% TFE, resulted in a significant increase in fluorescence intensity and a hypsochromic (blue) shift of ~8 nm. This shift indicates the binding of ANS to newly formed hydrophobic surfaces and likely involves electrostatic interactions with charged amino acid residues, such as lysine and arginine. Ion pairing with these residues and the ANS sulfonate group lowers the rate constant for intermolecular charge transfer, thus increasing fluorescence emission [66]. These findings demonstrate that even at relatively low concentrations, TFE effectively destabilizes the tertiary structure of A1AT, causing partial unfolding and exposing hydrophobic clusters. This process can be easily monitored through ANS binding. Incubating A1AT with just 20% TFE results in a marked increase in ANS fluorescence intensity compared to 10% TFE, highlighting the significant impact of TFE on protein structure. The modest rise at 10% TFE likely indicates initial disruption, producing an intermediate state in which hydrophobic residues are not fully exposed, resembling the early stages of a molten globule-like conformation. At 20% TFE, the substantial increase in fluorescence and blue shift confirms the formation of a molten globule-like structure with widespread exposure of hydrophobic regions, underscoring TFE’s potent destabilizing effect [65].

At higher solvent concentrations, a continuous loss of ANS fluorescence was observed. This decrease can be attributed to solvent-mediated molecular reorganization, in which partially folded intermediates interact and cross-link, conducive to the onset of aggregate formation [67]. The comparative ANS fluorescence profile of A1AT incubated with 0–70% TFE (Figure 4B) establishes the existence of discrete conformational species throughout the course of unfolding, before total aggregation. The modulatory influence of polyphenols on these structural transitions was also investigated (Figure 4C). Pre-incubation of A1AT with increasing concentrations of the compounds AMF and TF strongly attenuated ANS fluorescence in a dose-dependent fashion compared to solvent-treated controls. This attenuation indicates that both molecules restrict solvent-induced unfolding, thereby diminishing the exposure of hydrophobic patches and reducing the likelihood of aggregation. Of the two, AMF showed higher protective efficacy, reflecting a greater capacity to stabilize the protein and withstand TFE-induced conformational destabilization.

While intrinsic fluorescence and ANS binding strongly suggest the formation of a molten globule-like intermediate, these observations primarily reflect changes in tertiary packing and surface hydrophobicity. Definitive characterization of molten globule states also requires secondary structure analysis, which was beyond the scope of the present study.

### 4.5. Turbidity Assay

**Figure 5:**
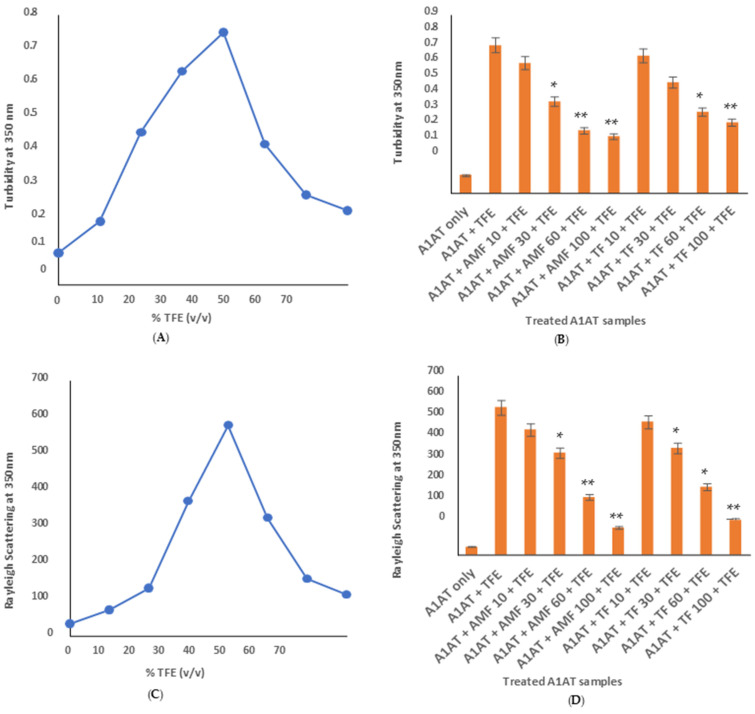
Turbidity and Rayleigh scattering measurements of the native and treated A1AT: (**A**) A1AT was incubated with increasing concentration (10–70% *v*/*v*) of TFE, and then the absorbance was recorded for native and TFE-treated A1AT samples at 350 nm. (**B**) Effect of varying concentrations of polyphenols AMF and TF on the turbidity of TFE incubated A1AT. Four different concentrations (10, 30, 60, and 100 µM) of both AMF and TF were used for the study. The TFE concentration used as a control is 40% (*v*/*v*). (**C**) A1AT was incubated with increasing concentrations (10–70% *v*/*v*) of TFE, and then the samples were excited at 350 nm, and the emission spectra were recorded from 300 to 400 nm. Peaks at 350 nm were plotted for the various TFE concentrations. (**D**) Effect of varying concentrations of polyphenols AMF and TF on the Rayleigh scattering of TFE incubated A1AT. Four different concentrations (10, 30, 60, and 100 µM) of both AMF and TF were used for the study. The TFE concentration used as a control is 40% (*v*/*v*). Statistical significance was assessed using one-way ANOVA followed by Tukey’s multiple comparisons test. Comparisons are shown relative to the A1AT + TFE group unless otherwise indicated (* *p* < 0.05, ** *p* < 0.01).

To confirm the fluorescence and ANS binding results indicating aggregation, turbidity measurements were conducted. As shown in Figure 5A, A1AT absorbance at 450 nm increased steeply from 20% TFE onward and peaked at 40% TFE. This rise indicates that aggregation is triggered at about 20% TFE and reaches maturity at 40% TFE. At elevated TFE concentrations, turbidity decreased progressively, eventually stabilizing at high solvent levels. The decrease in absorbance above 40% TFE could be due to solvent-induced solubilization or to structural rearrangements that reduce light scattering, even in the presence of unfolded conformers. The polyphenol-mediated protection against solvent-induced aggregation was also assessed (Figure 5B). When A1AT was pre-incubated with increasing concentrations of AMF and TF, followed by challenge with 40% TFE, a dose-dependent reduction in turbidity was observed. This indicates that both molecules successfully inhibited aggregate formation by stabilizing the protein in non-aggregating conformers. Between the two, AMF showed greater efficacy than TF, highlighting its stronger anti-aggregation activity. These findings validate the assumption that polyphenols, and more so AMF, can serve as structural stabilizers that inhibit solvent-induced aggregation pathways of A1AT.

The onset of turbidity at 20% TFE does not rule out the presence of molten globule-like species at this concentration. Rather, it suggests coexistence of partially folded intermediates and early oligomeric species, a pattern commonly observed during protein aggregation. The ANS fluorescence maximum at 20% TFE supports the predominance of partially folded intermediates, whereas turbidity indicates the onset of intermolecular association.

### 4.6. Rayleigh Scattering Measurement

Rayleigh scattering analysis was used to further analyze solvent-induced A1AT aggregation (Figure 5C). An enhancement of scattering intensity up to 40% TFE was observed, indicating the formation of protein aggregates upon treatment with TFE. At higher solvent concentrations (50–70% TFE), a significant decrease in scattering was observed, suggesting destabilization or disassembly of aggregates. Experimental work and simulation studies have indicated that fluorinated alcohols, such as TFE, cluster near protein surfaces, pushing out water and forming a low-dielectric environment conducive to intrapeptide hydrogen bonding. This preferential solvation stabilizes secondary structural components (e.g., α-helices), thus minimizing the propensity for oligomerization. The modulatory action of polyphenols on A1AT aggregation was also determined by Rayleigh scattering (Figure 5D). Pretreatment of A1AT with higher concentrations of AMF and TF considerably decreased scattering intensity in a dose-dependent manner as compared with solvent-treated controls. This suggests that both compounds efficiently reduced aggregation by stabilizing A1AT against solvent-mediated misfolding. As in previous assays, AMF exhibited more potent anti-aggregation activity than TF, underscoring its greater ability to maintain protein structural integrity under destabilizing conditions.

### 4.7. ThT Fluorescence Assay

**Figure 6:**
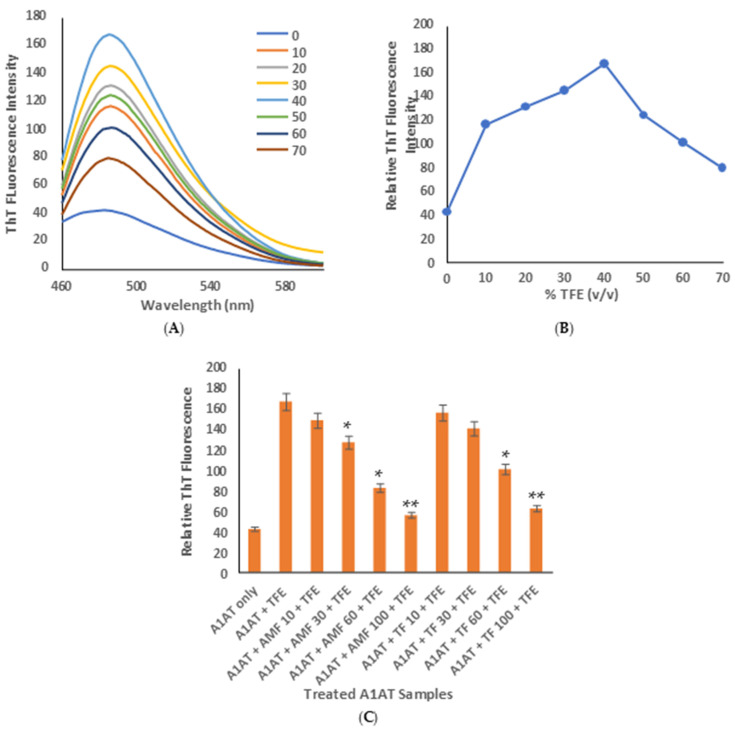
ThT fluorescence measurement of native and treated A1AT: (**A**) ThT fluorescence spectra of native and TFE (10–70% *v*/*v*) treated A1AT. (**B**) Relative ThT fluorescence pattern of native and TFE-treated A1AT. Peaks achieved on the respective TFE concentrations were plotted. (**C**) The effect of increasing concentrations of polyphenols, AMF, and TF on TFE-induced structural and conformational alterations in A1AT, as monitored by ThT fluorescence. Four different concentrations (10, 30, 60, and 100 µM) of both AMF and TF were used for the study. The TFE concentration used as the control is 40% (*v*/*v*). Statistical significance was assessed using one-way ANOVA followed by Tukey’s multiple comparisons test. Comparisons are shown relative to the A1AT + TFE group unless otherwise indicated (* *p* < 0.05, ** *p* < 0.01).

The appearance of amyloid fibrils within TFE-treated A1AT samples was determined by the ThT fluorescence assay. ThT is a benzothiazole dye that shows increased fluorescence (~485 nm) when it binds to the repetitive β-sheet-rich structures inherent in amyloid fibrils, as the loss of rotational freedom around its central C–C bond enhances quantum yield [68]. Native A1AT exhibited very weak ThT fluorescence, consistent with its non-amyloidogenic nature (Figure 6A). Conversely, 10% and 20% TFE-treated samples showed a moderate increase in ThT fluorescence, indicative of nascent aggregation. At 30% TFE, fluorescence intensity continued to rise, reflecting the accumulation of intermediate aggregates. The highest signal was observed at 40% TFE, depicting the growth of mature fibrillar structures (Figure 6B). TFE contribution alone to ThT fluorescence was subtracted, and spectra are background-subtracted. Collectively, these findings verify that solvent stress triggers amyloid-like aggregation of A1AT, with 40% TFE generating copious mature fibrils. The potential of polyphenols to block fibril formation was then assessed by ThT fluorescence in A1AT samples incubated with 40% TFE (Figure 6C). Pre-incubation with AMF and TF resulted in concentration-dependent reduction in fluorescence intensity. At 100 µM, both polyphenols almost completely quenched ThT fluorescence to native A1AT levels, indicating strong inhibition of fibril formation. AMF was found to be more potent as an anti-amyloidogenic agent than TF, especially at lower concentrations, indicating greater efficacy in inhibiting amyloid fibril formation. The IC_50_ values were estimated at approximately 40 µM and 50 µM for AMF and AT, respectively, based on interpolation of dose–response data at concentrations flanking the half-maximal inhibition. The comparative analysis of the two compounds reveals a modest but consistent difference in inhibitory potency toward TFE-induced A1AT activity. AMF exhibited a lower IC_50_ (~40 µM) than AT (~50 µM), indicating greater potency under the tested conditions. Both compounds demonstrated a clear dose-dependent inhibitory trend; however, the shift in IC_50_ suggests differences in their interaction efficiency or binding affinity with the target system. Despite this observed variation, the IC_50_ values were derived using interpolation due to the limited number of concentration points and should therefore be interpreted as approximate estimates rather than definitive quantitative measures. Nonetheless, the relative difference in IC_50_ values supports the conclusion that the first compound exerts a stronger inhibitory effect than the second within the concentration range examined.

## 5. Molecular Dynamics Simulation

### 5.1. Analysis of RMSD and RMSF

**Figure 7:**
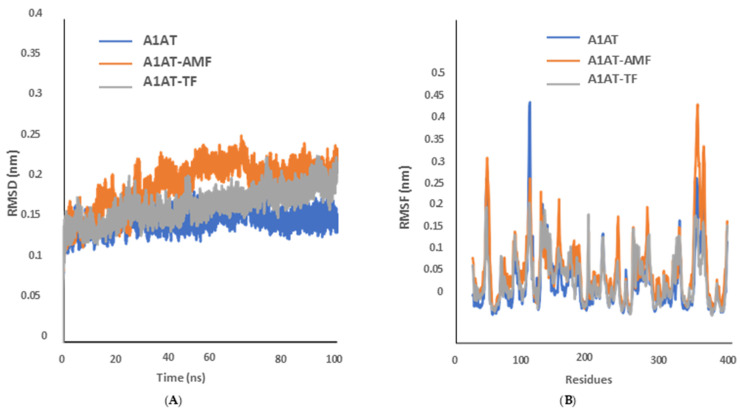
(**A**) RMSD of the backbone of A1AT alone, A1AT-AMF complex, and A1AT-TF complex during the entire simulation period. (**B**) RMSF of Cα atoms of all the residues of the backbone of A1AT alone, A1AT-AMF complex, and A1AT-TF complex.

The MD trajectories were first assessed by calculating the root mean square deviation (RMSD) of each system from its initial conformation. RMSD analysis helps to explore the structural stability of proteins and protein–ligand complexes throughout simulations [69]. As shown in Figure 7A, all systems exhibited significant fluctuations during the first 30 ns, after which they settled into equilibrium, indicating that the trajectories were well stabilized by this time. The average RMSD for A1AT in isolation was 0.149 nm, while the A1AT–AMF and A1AT–TF complexes had average RMSD values of 0.195 nm and 0.168 nm, respectively. The slightly higher RMSD observed for the A1AT-AMF and A1AT-TF complexes relative to unbound A1AT may reflect ligand-induced conformational adjustments rather than destabilization. Such local flexibility could facilitate stabilizing interactions that hinder aggregation-prone conformational transitions. Importantly, this increased RMSD does not correlate with structural instability, as experimentally reduced aggregation supports functional stabilization rather than unfolding. The root mean square fluctuation (RMSF) of the Cα atoms was determined in order to better investigate structural plasticity (Figure 7B). All the residues showed fluctuations of less than 0.1 nm, consistent with the systems’ overall stability. Localized maxima in RMSF were mainly linked with loop and coil regions of A1AT, which are naturally flexible in the aqueous phase. Furthermore, the atomic RMSF of the ligands was also analyzed to measure their conformational dynamics, as shown in Figure S1 (Supplementary Information). AMF exhibited lower fluctuations, indicating relatively stable binding, whereas TF showed greater flexibility, possibly due to more rotatable bonds and dynamic interactions within the binding pocket.

### 5.2. Analysis of Structure Compactness, Physicochemical Parameters, and Secondary Structure

**Figure 8:**
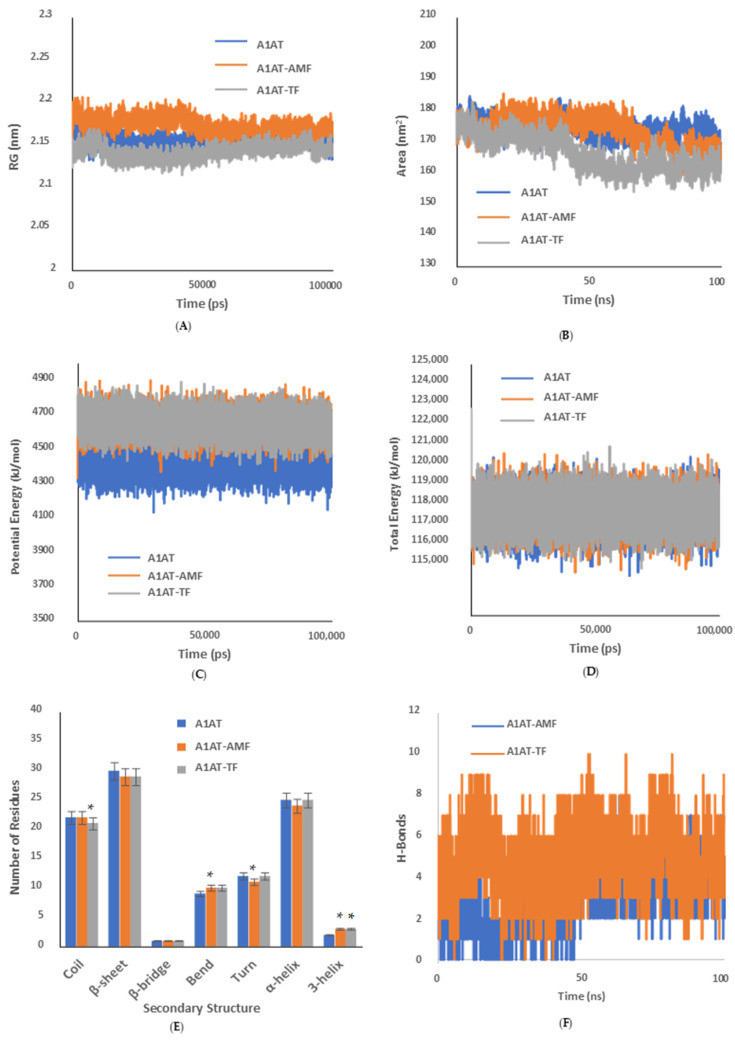
(**A**) Radius of gyration (RG) of the backbone of A1AT alone, A1AT-AMF complex, and A1AT-TF complex. (**B**) Solvent-accessible surface area (SASA) of A1AT alone, A1AT-AMF complex, and A1AT-TF complex as a function of simulation time. (**C**) Potential energy of A1AT alone, A1AT-AMF complex, and A1AT-TF complex over 100 ns MD simulation. (**D**) Total energy of A1AT alone, A1AT-AMF complex, and A1AT-TF complex over 100 ns MD simulation. (**E**) Secondary structure of A1AT alone, A1AT-AMF complex, and A1AT-TF complex calculated using the DSSP method in gromacs. (**F**) Number of hydrogen bonds formed between ligands (AMF and TF) and A1AT over a 100 ns MD simulation. Statistical significance was assessed using one-way ANOVA followed by Tukey’s multiple comparisons test. Comparisons are shown relative to the A1AT (* *p* < 0.05).

The radius of gyration (RG), which is the root mean square distance of a group of atoms from their shared center of mass, weighted by mass, is commonly used to assess the structural tightness and stability of proteins during molecular dynamics (MD) simulations. Globular proteins usually exhibit diminished RG fluctuations compared with unfolded or stretched conformations [70,71]. RG profiles of A1AT and its ligand-bound structures are shown in Figure 8A. The average RG of A1AT alone was 2.154 nm, while the A1AT–AMF and A1AT–TF complexes had average values of 2.173 nm and 2.141 nm, respectively. These findings show that ligand binding did not cause any significant conformational expansion, further reflecting the structural stability of the complexes. To support this analysis, the solvent-accessible surface area (SASA) for each system was computed (Figure 8B). A1AT alone had an average SASA of 174.182 nm^2^, while the SASA for the A1AT–AMF and A1AT–TF complexes were 173.634 nm^2^ and 166.415 nm^2^, respectively. The subtle differences in SASA values relative to the unbound protein indicate minimal changes in solvent exposure, further affirming the compact and stable nature of all systems under simulation conditions.

Moreover, the physicochemical stability of A1AT and its complexes under water-like conditions was assessed by tracking the potential and total energies along the trajectories (Figure 8C,D). The potential and total energy profiles of the complexes were stable and very similar to those of the free protein, confirming the absence of harmful energetic perturbations. Overall, these parameters support the global structural stability of A1AT and its ligand-bound forms under physiologically relevant conditions [72]. The impact of ligand binding on the structural stability of A1AT was determined by examining its secondary structure in the free and complexed states. The distribution of secondary structure elements for each system is shown in Figure 8E. For the free protein, the ratios of coil, β-sheet, β-bridge, bend, turn, α-helix, and 3_10_-helix were 22%, 30%, 1%, 9%, 12%, 25%, and 2%, respectively. Similar distributions were found for A1AT–AMF and A1AT–TF complexes, with no significant changes in the structural motifs upon ligand association. These results show that binding with AMF and TF does not disrupt the secondary structure of A1AT, thus further supporting the overall structural stability of the simulated systems.

### 5.3. Analysis of Hydrogen Bonds and Binding Energies

Interactions between A1AT and the ligands were studied by analyzing hydrogen bond formation along MD trajectories. The average hydrogen bond count across the A1AT–AMF and A1AT–TF complexes was 3.060 and 4.773, respectively (Figure 8F), indicating that TF formed more hydrogen bonds with A1AT than AMF. This is consistent with the docking, which showed that TF had more widespread hydrogen-bonding contacts with the protein. These differences may also be due to competition and restructuring water molecules at the binding site, which can temporarily alter hydrogen-bond occupancy [73,74].

To further evaluate binding affinities, molecular mechanics Poisson–Boltzmann surface area (MM-PBSA) calculations were carried out. For each analysis, 100 snapshots were taken at 1 ns intervals from each complex trajectory. Protein–ligand interactions are largely governed by non-covalent forces, such as electrostatic, hydrophobic, van der Waals, and hydrogen-bonding contributions, which can stabilize or destabilize binding [75]. The binding free energy decomposition showed that electrostatic and van der Waals interactions were predominant for ligand binding to A1AT, while SASA energy contributed less (Table 1). Polar solvation energy, however, showed positive values, indicating unfavorable conditions for complex formation. The estimated total binding free energy was −99.872 kJ/mol for AMF and −40.868 kJ/mol for TF, indicating better affinity of AMF towards A1AT. Residue-level energy decomposition also revealed the major contributors to ligand binding (Table 2). For A1AT–AMF complex, residues Tyr-38, Ser-45, Ser-47, Thr-48, Asn-49, His-209, Asp-211, Gln-212, Lys-233, Leu-254, Asp-256, Glu-257, Gly-258, Leu-260, Gln-261, His-262, Glu-264, Pro-369, Phe-370, Val-389, and Gln-393 made major contributions to stabilization. For the A1AT–TF complex, it was seen that Tyr-297, Gly-295, Thr-294, Ile-50, Thr-48, Leu-41, Val-333, Lys-331, Val-170, Leu-172, Ile-293, and Thr-296 were the important contributors. Interestingly, some of these residues had positive polar energy values, suggesting that their polar solvation lowered the overall binding energy despite the stabilizing contributions of van der Waals and electrostatic interactions.

### 5.4. Principal Component Analysis

**Figure 9:**
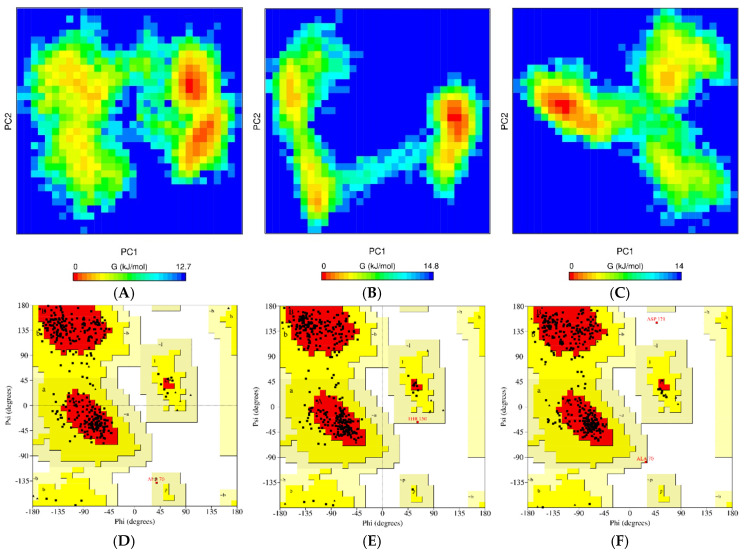
The free energy landscapes of (**A**) A1AT alone, (**B**) A1AT-AMF complex, and (**C**) A1AT-TF complex. Ramachandran plots of energy minima structure of (**D**) A1AT alone, (**E**) A1AT-AMF complex, and (**F**) A1AT-TF complex.

Principal component analysis (PCA) is another commonly used statistical method for examining proteins’ collective motions by reducing data dimensionality while retaining vital information, as captured by the eigenvectors [76]. PCA was utilized to analyze differences in the structural flexibility of A1AT and its ligand-bound complexes. Eigenvector projection showed that both free A1AT and the complexes occupied distinct regions of conformational space, with wider distributions reflecting greater structural flexibility. To better define conformational dynamics, free energy landscapes were built from the major components (Figure 9A–C). All systems converged to energy minima throughout the simulations, validating thermodynamic stability, although variations in the locations of the minima indicated changes in conformational states. The lowest-energy minima were then used to obtain representative structures from the trajectories, which were assessed using Ramachandran plot analysis (Figure 9D–F). Distribution of residue positions among Ramachandran plot categories is depicted in Table S2 (Supplementary Information). Across all systems, most residues were found within the most favored and additionally allowed regions of the Ramachandran plots, with essentially zero or negligible residues in the generously allowed or disallowed regions. This distribution reflects the conformational stability of both free A1AT and its complexes, suggesting that ligand binding did not trigger improper structural distortions during the simulation.

## 6. Discussion

Proteins, with their remarkable sequence heterogeneity, possess the ability to spontaneously fold into precise three-dimensional structures that underlie their functions [77]. Under optimal conditions, cellular quality-control mechanisms, such as molecular chaperones and proteostasis networks, oversee protein folding and remove misfolded proteins before they cause harm [78]. However, some proteins evade these defenses and engage in aberrant self-association, leading to aggregate formation, disruption of cellular integrity, and, in extreme cases, cell death. Misfolding and aggregation form the basis of the pathology of numerous neurodegenerative diseases, such as Alzheimer’s, Parkinson’s, Huntington’s, and prion diseases, all of which are marked by the formation of abnormal protein assemblies [79]. Depending on their structural arrangement, these aggregates may be classified as ordered fibrillar species (amyloid) or disordered, amorphous aggregates, such as inclusion bodies [80,81]. Amyloid fibrils are characterized by having a very regular and dense β-sheet structure, which binds distinctively to dyes like ThT, whereas amorphous aggregates do not have this structural regularity. Therefore, elucidating the mechanisms underlying the formation of these aggregates is vital for understanding disease occurrence and developing selective therapeutic interventions. Unraveling the molecular mechanisms of protein aggregation and amyloid fibril assembly remains a grand challenge in protein science, with numerous mechanistic aspects still poorly understood [82]. A hallmark feature of amyloid fibrils and protein aggregates is a conserved cross-β core structure that occurs irrespective of the primary amino acid sequence of the precursor protein [83]. Aggregation often initiates when native proteins inhabit partially folded or intermediate conformations. These metastable phases typically expose occluded hydrophobic residues, thereby encouraging intermolecular interactions that drive the formation of cross-linked assemblies.

In this study, the fluorinated alcohol TFE was used as a model denaturant to investigate the conformational stability and tendency to aggregate of A1AT. The choice of TFE is particularly relevant because it promotes aggregation via molten globule-like intermediates rather than complete denaturation, making it suitable for studying early events in amyloidogenesis and for screening anti-aggregation compounds [48]. Incubation of A1AT with TFE over the range 0–20% (*v*/*v*) brought about structural perturbations, leading to the production of partially folded intermediates and molten globule-like structures, both of which had a strong tendency for fibrillation. At intermediate levels (30–40% *v*/*v*), A1AT displayed conformational changes, which may be due to a loss of α-helical content and a gain of β-sheet structures, typical of aggregation-prone conformations [80]. A possible explanation is that within this concentration window, the number of TFE molecules is insufficient to fully solvate and destabilize hydrophobic clusters in A1AT, leaving these aggregation-prone areas exposed and able to associate intermolecularly. Functionally, the antitrypsin activity assay showed a gradual reduction in inhibitory activity as TFE concentration increased, reaching a minimum at about 40% (*v*/*v*), which correlates with the maximum aggregation and structural deviation from the native conformation. These findings not only explain the conformational plasticity of A1AT but also highlight the subtle interplay between folding, misfolding, and aggregation that regulates protein homeostasis. Comparisons within each assay were performed at fixed TFE concentrations to ensure internal consistency.

Intrinsic fluorescence spectroscopy yielded further confirmation of A1AT structural perturbations with TFE. At 30% and 40% TFE, there was a large reduction in fluorescence intensity, accompanied by a red shift in λmax, indicating modifications in the local environment of the aromatic residues and confirming conformational destabilization. These transitions were further supported by the increased turbidity and Rayleigh scattering under identical conditions, as expected with the formation of bigger cross-linked assemblies or aggregates. To learn more about the intermediate states of A1AT, we used 8-anilinonaphthalene-1-sulfonic acid (ANS), a widely used hydrophobic probe that preferentially binds to molten globule-like intermediates rather than to native or completely denatured proteins. Maximum fluorescence of ANS was observed at 20% TFE, strongly indicating that A1AT exists in a molten globule-like state under these conditions. The increased signal can be assigned to the exposure of hydrophobic residues that become accessible to the dye in this intermediate state. By contrast, the decrease in ANS fluorescence at 30% and 40% TFE suggested that the protein aggregates, likely due to the collapse and coalescence of partially structured intermediates into higher-order fibrillar structures, limit dye penetration to buried hydrophobic patches. This event is likely driven by the transition of α-helical components into extended β-sheet structures upon TFE exposure [83]. These aggregates were found to be fibrillogenic by ThT fluorescence assays, a well-established marker for amyloid fibrils. The binding of ThT showed a significant increase in fluorescence intensity at 30% and 40% TFE compared to the native protein, clearly establishing the presence of amyloid-like fibrils. However, time-resolved kinetic analysis is an important direction for future work. We acknowledge that both intrinsic Trp fluorescence and ANS binding are sensitive to TFE concentration. Accordingly, all comparative analyses were performed at fixed TFE concentrations to isolate protein conformational effects from solvent contributions.

Together, these spectroscopic and dye-binding experiments uncover a stepwise A1AT aggregation pathway in the presence of TFE, from an intermediate molten globule-like conformation (20% TFE) to amyloid fibril-like structures (30–40% TFE). Although ThT fluorescence indicates a marked reduction in amyloid fibril formation in the presence of AMF and TF, turbidity and Rayleigh scattering data suggest that some larger species may still be present, albeit reduced. This indicates that these polyphenols may not entirely eliminate aggregation but rather redirect the pathway toward the formation of less ordered or less ThT-reactive aggregates. Such redirection of aggregation pathways has been reported for several polyphenolic inhibitors and may represent a protective mechanism by preventing the formation of toxic amyloid fibrils [84]. Further characterization using techniques such as TEM, AFM, or DLS would be valuable to distinguish between fibrillar and amorphous species and will be pursued in future studies.

It should be noted that fluorescence-based techniques alone cannot conclusively establish a molten globule state, as such assignments ideally require far-UV CD or FTIR analysis to confirm retention of secondary structure. Therefore, we refer to the observed intermediate as a molten globule-like state, based on combined fluorescence and ANS-binding behavior, consistent with previously reported spectroscopic signatures of molten globules [85].

AMF, a naturally occurring biflavonoid found in large quantities in certain plants, such as Ginkgo biloba and *Selaginella tamariscina*, has attracted considerable attention for its potent anti-aggregation and anti-amyloidogenic activity [86]. These activities are especially important in relation to protein misfolding disorders like Alzheimer’s disease, Parkinson’s disease, Huntington’s disease, and systemic amyloidosis [87]. In addition, AMF has been associated with anti-oxidative, anti-inflammatory, and neuroprotective properties, which collectively contribute to its therapeutic potential in the fight against amyloid-related neurodegeneration [88]. TF, polyphenolic pigments formed during black tea fermentation, are potent inhibitors of amyloid formation, effectively blocking aggregation of amyloid-β (Aβ) and α-synuclein, proteins involved in Alzheimer’s and Parkinson’s diseases. In addition to their anti-amyloidogenic activity, theaflavins demonstrate strong antiplatelet and antithrombotic effects in vitro and in vivo, highlighting their potential to prevent vascular complications associated with neurodegenerative disorders [89,90]. However, there are some limitations: AMF exhibits poor aqueous solubility, extensive first-pass metabolism, and low systemic exposure, with most of the absorbed AMF appearing as conjugated metabolites [91]. TF has limited bioavailability due to moderate absorption and metabolism by gut microbiota [92].

To better understand the structural interactions of A1AT with the ligands AMF and TF, molecular docking and molecular dynamics simulations were conducted. Molecular docking and molecular dynamics simulations were performed under aqueous conditions, without TFE, to examine the intrinsic binding stability and structural effects of polyphenols on native A1AT. Experimental aggregation induced by TFE was evaluated using simulations to determine whether AMF and TF can stabilize A1AT under physiologically relevant conditions and prevent structural destabilization prior to aggregation [68]. Thus, the simulations complement the experimental data by demonstrating that polyphenol binding intrinsically stabilizes the A1AT structure, supporting the observed anti-aggregation effects under solvent-stress conditions. While MD simulations provide insight into secondary-structure stability, experimental validation using far-UV circular dichroism or FTIR spectroscopy would be required to directly monitor changes in α-helical and β-sheet structures during TFE-induced aggregation and to assess their modulation by polyphenols [68]. This represents an important direction for future investigation.

The analyses showed stable binding of both ligands to the protein, indicating their potential to exert anti-aggregating effects by stabilizing the binding sites while maintaining the general structural integrity of A1AT. The strong inhibition by AMF indicates a better interaction with A1AT, which was confirmed using in silico methods—molecular docking and MD simulation. This may include stabilizing partially folded intermediates and preventing β-sheet stacking, which promotes fibril elongation. These findings further emphasize AMF as a more effective anti-fibrillogenic and anti-aggregating agent compared to TF under the conditions used here. The present work focuses on steady-state and end-point biophysical characterization of A1AT aggregation and its modulation by polyphenols. Detailed kinetic analysis of amyloid formation and direct morphological visualization by electron microscopy would provide deeper mechanistic insight into fibril nucleation, elongation, and inhibition. Although such analyses are beyond the scope of this study, future work will include time-resolved ThT kinetics and EM-based structural validation of fibril inhibition by AMF and TF. Future studies using structural and thermodynamic analyses will also be critical to uncover the molecular mechanism(s) by which AMF and TF regulate A1AT misfolding and evaluate their potential as lead scaffolds for anti-aggregation strategies as protein aggregation modulators.

## 7. Conclusions

This study presents biophysical insights into TFE-induced alpha-1-antitrypsin misfolding and aggregation, establishing a pathway from molten globule-like intermediates to β-sheet-enriched amyloid fibrils at 30–40% TFE. Employing complementary spectroscopic, light-scattering, and dye-binding methods, we showed that the polyphenols AMF and TF effectively inhibit fibril formation and aggregation, with AMF exhibiting a more potent protective effect than TF. Docking and MD simulations in aqueous solution demonstrate that both compounds form stable complexes with A1AT without disrupting its secondary structure, and MM-PBSA analysis reveals a more favorable overall binding free energy for AMF, in line with its greater experimental potency. These observations suggest that natural polyphenols are promising scaffolds for designing therapeutic agents against protein misfolding and amyloidogenesis and have potential as lead molecules for anti-aggregation strategies in the treatment of A1AT deficiency and other amyloid-based diseases. At the same time, the use of TFE as a non-physiological cosolvent, the lack of kinetic ThT measurements and direct fibril morphology, and the limited in vivo bioavailability of AMF and TF underscore the need for future studies incorporating detailed secondary structure analysis, morphology by electron microscopy, time-resolved aggregation kinetics, and cellular or animal models of A1AT deficiency.

## Figures and Tables

**Table 1:** Binding free energy (kJ/mol) for the interaction of polyphenols AMF and TF with A1AT calculated using MM-PBSA analysis for 100 snapshots of MD simulation.

Energy (kJ/mol)	A1AT-AMF	A1AT-TF
ΔE_vdW_	−204.454 +/− 1.450	−157.096 +/− 1.814
ΔE_ele_	−97.258 +/− 1.446	−114.222 +/− 2.111
ΔE_PSE_	221.775 +/− 1.753	249.750 +/− 2.791
ΔE_SASA_	−19.925 +/− 0.106	−19.363 +/− 0.176
ΔE_BE_	−99.872 +/− 1.584	−40.868 +/− 1.724

ΔE_vdW_: van der Waal energy, ΔE_ele_: Electrostatic energy, ΔE_PSE_: Polar solvation energy, ΔE_SASA_: Solvent-accessible surface area energy, ΔE_BE_: Binding energy.

**Table 2:** MM, apolar, polar, and total binding energies of the major energy contributing residues of A1AT for their interaction with AMF and TF.

Residues	MM Energy	Polar Energy	Apolar Energy	Total Energy
A1AT-AMF				
Tyr-38	−0.0086 ± 0.0147	−0.6641 ± 0.0195	0 ± 0	−0.6736 ± 0.0237
Ser-45	−0.6796 ± 0.0311	−0.4264 ± 0.0863	−0.0038 ± 0.0025	−1.1101 ± 0.0835
Ser-47	−6.5807 ± 0.2912	4.4741 ± 0.2688	−0.794 ± 0.037	−2.9042 ± 0.2306
Thr-48	−0.9891 ± 0.0443	−1.2862 ± 0.0596	−0.0007 ± 0.0007	−2.2741 ± 0.0737
Asn-49	−0.2519 ± 0.0133	−0.2802 ± 0.0228	0 ± 0	−0.5317 ± 0.0214
His-209	−4.0881 ± 0.2945	2.7719 ± 0.3089	−0.2055 ± 0.0105	−1.5314 ± 0.2169
Asp-211	−15.1262 ± 0.4821	10.589 ± 0.5286	−0.448 ± 0.0196	−4.993 ± 0.3827
Gln-212	−6.347 ± 0.2016	3.1273 ± 0.2872	−0.3448 ± 0.014	−3.5685 ± 0.2653
Lys-233	−0.735 ± 0.0219	0.1319 ± 0.0098	0 ± 0	−0.6037 ± 0.0196
Leu-254	−0.3749 ± 0.0118	−0.2047 ± 0.0255	0 ± 0	−0.581 ± 0.026
Asp-256	0.9572 ± 0.1199	−4.2317 ± 0.1461	−0.0309 ± 0.0046	−3.3058 ± 0.1603
Glu-257	0.1035 ± 0.1308	−1.8705 ± 0.1854	−0.1089 ± 0.0062	−1.8721 ± 0.1089
Gly-258	−10.1734 ± 0.147	9.0801 ± 0.1142	−0.6299 ± 0.0143	−1.7122 ± 0.1672
Leu-260	−10.9353 ± 0.1535	5.6838 ± 0.0868	−0.4169 ± 0.0115	−5.678 ± 0.1566
Gln-261	−11.9414 ± 0.2916	8.3704 ± 0.2766	−1.1886 ± 0.027	−4.7716 ± 0.1746
His-262	−0.802 ± 0.0238	0.1227 ± 0.0577	0 ± 0	−0.6794 ± 0.0475
Glu-264	−22.3134 ± 0.3433	11.217 ± 0.5166	−0.1649 ± 0.0099	−11.2405 ± 0.5096
Pro-369	−8.7186 ± 0.1205	4.1571 ± 0.1087	−0.6035 ± 0.0151	−5.1624 ± 0.1583
Phe-370	−0.6434 ± 0.0105	−0.1753 ± 0.0163	0 ± 0	−0.8183 ± 0.0186
Val-389	−7.377 ± 0.1203	2.5243 ± 0.1018	−0.5935 ± 0.012	−5.4493 ± 0.1256
Gln-393	−1.0174 ± 0.0508	−0.5222 ± 0.0554	0 ± 0	−1.5371 ± 0.0511
A1AT-TF				
Tyr-297	−8.7703 ± 0.2424	4.9072 ± 0.1828	−0.523 ± 0.02	−4.3915 ± 0.1632
Gly-295	−6.073 ± 0.0985	3.498 ± 0.1014	−0.2037 ± 0.0077	−2.7779 ± 0.0971
Thr-294	−7.9491 ± 0.3218	6.4636 ± 0.2925	−0.8214 ± 0.0293	−2.3033 ± 0.2958
Ile-50	−2.5258 ± 0.1508	0.5555 ± 0.0567	−0.0996 ± 0.0137	−2.0679 ± 0.1181
Thr-48	−5.8656 ± 0.2823	4.6644 ± 0.2345	−0.576 ± 0.0229	−1.7653 ± 0.1791
Leu-41	−1.2706 ± 0.1138	0.4171 ± 0.0707	−0.0777 ± 0.0131	−0.9306 ± 0.09
Val-333	−0.4867 ± 0.0438	−0.3637 ± 0.0414	−0.0136 ± 0.0035	−0.8655 ± 0.0489
Lys-331	−0.4331 ± 0.2745	−0.2523 ± 0.2396	−0.0085 ± 0.0042	−0.701 ± 0.1654
Val-170	−0.3539 ± 0.0541	−0.3317 ± 0.0652	0 ± 0	−0.6884 ± 0.038
Leu-172	−4.2676 ± 0.2436	3.7891 ± 0.173	−0.0823 ± 0.0099	−0.5481 ± 0.2191
Ile-293	−0.6043 ± 0.0345	0.0658 ± 0.0423	−0.008 ± 0.0025	−0.5442 ± 0.0325
Thr-296	−23.3423 ± 0.3581	20.8981 ± 0.2108	−1.4705 ± 0.0164	−3.9211 ± 0.3284

## Data Availability

The original contributions presented in this study are included in the article/Supplementary Materials. Further inquiries can be directed to the corresponding author.

## Supplementary Materials

The following supporting information can be downloaded at https://www.mdpi.com/article/10.3390/biomedicines14061310/s1, Figure S1: RMSF of each atom of ligands AMF and TF during 100 ns MD simulation; Table S1: Molecular docking-based screening data and affinity values (binding free energies in kcal/mol) of alpha-1 antitrypsin (A1AT) with phytocompounds; Table S2: The number of residues falling into various categories across all energy minima structures for A1AT, A1AT-AMF, and A1AT-TF complex.

## References

[1] Chiti F., Dobson C.M. (2017). Protein misfolding, amyloid formation, and human disease: A summary of progress over the last decade. Annu. Rev. Biochem..

[2] Malik S., Siddiqi M.K., Majid N., Masroor A., Moasfar Ali S., Khan R.H. (2020). Unravelling the inhibitory and cytoprotective potential of diuretics towards amyloid fibrillation. Int. J. Biol. Macromol..

[3] Schroeder W.T., Miller M.F., Woo S.L., Saunders G.F. (1985). Chromosomal localization of the human alpha 1-antitrypsin gene (PI) to 14q31–32. Am. J. Hum. Genet..

[4] Hunt L.T., Dayhoff M.O. (1980). A surprising new protein superfamily containing ovalbumin, antithrombin-III, and alpha1-proteinase inhibitor. Biochem. Biophys. Res. Commun..

[5] Van Gent D., Sharp P., Morgan K., Kalsheker N. (2003). Serpins: Structure, function and molecular evolution. Int. J. Biochem. Cell Biol..

[6] Perlmutter D.H., Cole F.S., Kilbridge P., Rossing T.H., Colten H.R. (1985). Expression of the alpha 1-proteinase inhibitor gene in human monocytes and macrophages. Proc. Natl. Acad. Sci. USA.

[7] Mulgrew A.T., Taggart C., Lawless M.W., Greene C., Brantly M.L., O’Neill S.J., McElvaney N.G. (2004). Z 1-antitrypsin polymerizes in the lung and acts as a neutrophil chemoattractant. Chest.

[8] Molmenti E.P., Perlmutter D.H., Rubin D.C. (1993). Cell-specific expression of alpha 1-antitrypsin in human intestinal epithelium. J. Clin. Investig..

[9] Gabay C., Kushner I. (1999). Acute-phase proteins and other systemic responses to inflammation. N. Engl. J. Med..

[10] Bergin D.A., Reeves E.P., Hurley K., Wolfe R., Jameel R., Fitzgerald S., McElvaney N.G. (2014). The circulating proteinase inhibitor α-1 antitrypsin regulates neutrophil degranulation and autoimmunity. Sci. Transl. Med..

[11] McCarthy C., Saldova R., Wormald M.R., Rudd P.M., McElvaney N.G., Reeves E.P. (2014). The role and importance of glycosylation of acute phase proteins with focus on alpha-1 antitrypsin in acute and chronic inflammatory conditions. J. Proteome Res..

[12] Bashir A., Shah N.N., Hazari Y.M., Habib M., Bashir S., Hilal N., Banday M., Asrafuzzaman S., Fazili K.M. (2016). Novel variants of SERPIN1A gene: Interplay between alpha1-antitrypsin deficiency and chronic obstructive pulmonary disease. Respir. Med..

[13] Loebermann H., Tokuoka R., Deisenhofer J., Huber R. (1984). Human 1-proteinase inhibitor: Crystal structure analysis of two crystal modifications, molecular model and preliminary analysis of the implications for function. J. Mol. Biol..

[14] Ryu S.-E., Choi H.-J., Kwon K.-S., Lee K.N., Yu M.-H. (1996). The native strains in the hydrophobic core and flexible reactive loop of a serine protease inhibitor: Crystal structure of an uncleaved 1-antitrypsin at 2.7 Å. Structure.

[15] Ogushi F., Fells G.A., Hubbard R.C., Straus S.D., Crystal R.G. (1987). Z-type alpha 1-antitrypsin is less competent than M1-type alpha 1-antitrypsin as an inhibitor of neutrophil elastase. J. Clin. Investig..

[16] Krishnan B., Gierasch L.M. (2011). Dynamic local unfolding in the serpin alpha-1 antitrypsin provides a mechanism for loop insertion and polymerization. Nat. Struct. Mol. Biol..

[17] Tsutsui Y., Dela Cruz R., Wintrode P.L. (2012). Folding mechanism of the metastable serpin alpha1-antitrypsin. Proc. Natl. Acad. Sci. USA.

[18] Bashir A., Hazari Y., Pal D., Maity D., Bashir S., Singh L.R., Shah N.N., Fazili K.M. (2020). Aggregation of M3 (E376D) variant of alpha1-antitrypsin. Sci. Rep..

[19] Okigawa M., Hwa C.W., Kawano N., Rahman W. (1971). Biflavones in Selaginella species. Phytochemistry.

[20] Arwa P.S., Zeraik M.L., Ximenes V.F., da Fonseca L.M., Bolzani V.S., Silva D.H.S. (2015). Redox-active biflavonoids from Garcinia brasiliensis as inhibitors of neutrophil oxidative burst and human erythrocyte membrane damage. J. Ethnopharmacol..

[21] Abdallah H.M., Almowallad F.M., Esmat A., Shehata I.A., Abdel-Sattar E.A. (2015). Anti-inflammatory activity of flavonoids from Chrozophora tinctoria. Phytochem. Lett..

[22] Park N.H., Lee C.W., Bae J.H., Na Y.J. (2011). Protective effects of amentoflavone on Lamin A-dependent UVB-induced nuclear aberration in normal human fibroblasts. Bioorg. Med. Chem. Lett..

[23] Ndongo J.T., Issa M.E., Messi A.N., Mbing J.N., Cuendet M., Pegnyemb D.E., Bochet C.G. (2015). Cytotoxic flavonoids and other constituents from the stem bark of Ochna schweinfurthiana. Nat. Prod. Res..

[24] Coulerie P., Nour M., Maciuk A., Eydoux C., Guillemot J.C., Lebouvier N., Hnawia E., Leblanc K., Lewin G., Canard B. (2013). Structure-activity relationship study of biflavonoids on the Dengue virus polymerase DENV-NS5 RdRp. Planta Medica.

[25] Hwang I.S., Lee J., Jin H.G., Woo E.R., Lee D.G. (2012). Amentoflavone stimulates mitochondrial dysfunction and induces apoptotic cell death in Candida albicans. Mycopathologia.

[26] Zhang Z., Sun T., Niu J.G., He Z.Q., Liu Y., Wang F. (2015). Amentoflavone protects hippocampal neurons: Anti-inflammatory, antioxidative, and antiapoptotic effects. Neural Regen. Res..

[27] Zheng X.K., Liu C.X., Zhai Y.Y., Li L.L., Wang X.L., Feng W.S. (2013). Protection effect of amentoflavone in *Selaginella tamariscina* against TNF-induced vascular injure of endothelial cells. Acta Pharm. Sin..

[28] Chinese Pharmacopeia Commission (2015). Pharmacopoeia of the People’s Republic of China.

[29] Zhang J., Cai S., Li J., Xiong L., Tian L., Liu J., Huang J., Liu Z. (2016). Neuroprotective effects of theaflavins against oxidative stress-induced apoptosis in PC12 cells. Neurochem. Res..

[30] Wu D., Mei S., Duan R., Geng F., Wu W., Li X., Cheng L., Wang C. (2020). How black tea pigment theaflavin dyes chicken eggs: Binding affinity study of theaflavin with ovalbumin. Food Chem..

[31] Tong T., Liu Y.J., Kang J., Zhang C.M., Kang S.G. (2019). Antioxidant activity and main chemical components of a novel fermented tea. Molecules.

[32] Chakrabarty S., Nag D., Ganguli A., Das A., Ghosh D.D., Chakrabarti G. (2019). Theaflavin and epigallocatechin-3-gallate synergistically induce apoptosis through inhibition of PI3K/Akt signaling upon depolymerizing microtubules in HeLa cells. J. Cell. Biochem..

[33] Salimikia I., Aminnezhad S., Maghsoudloo M., Mirzania F. (2023). Anti-neurodegenerative, anticancer, anti-inflammatory, and antiobesity activities of theaflavin and its derivatives. Micro Nano Bio Asp..

[34] Teng Z., Guo Y., Liu X., Zhang J., Niu X., Yu Q., Deng X., Wang J. (2019). Theaflavin-3,3-digallate increases the antibacterial activity of beta-lactam antibiotics by inhibiting metallo-beta-lactamase activity. J. Cell. Mol. Med..

[35] Gothandam K., Ganesan V.S., Ayyasamy T., Ramalingam S. (2019). Antioxidant potential of theaflavin ameliorates the activities of key enzymes of glucose metabolism in high fat diet and streptozotocin-induced diabetic rats. Redox Rep..

[36] Peluso I., Serafini M. (2017). Antioxidants from black and green tea: From dietary modulation of oxidative stress to pharmacological mechanisms. Br. J. Pharmacol..

[37] Li R., Li X., Wu H., Yang Z., Fei L., Zhu J. (2019). Theaflavin attenuates cerebral ischemia/reperfusion injury by abolishing miRNA1283p-mediated Nrf2 inhibition and reducing oxidative stress. Mol. Med. Rep..

[38] Han X., Zhang J., Xue X., Zhao Y., Lu L., Cui M., Miao W. (2017). Theaflavin ameliorates ionizing radiation-induced hematopoietic injury via the NRF2 pathway. Free Radic. Biol. Med..

[39] Wang Q., Xie C., Xi S., Qian F., Peng X., Huang J., Tang F. (2020). Radioprotective effect of flavonoids on ionizing radiation-induced brain damage. Molecules.

[40] Karatas E., Bouchecareilh M. (2020). Alpha 1-Antitrypsin Deficiency: A Disorder of Proteostasis-Mediated Protein Folding and Trafficking Pathways. Int. J. Mol. Sci..

[41] Rao L., Xu Y., Reineke L.C., Bhattacharya A., Tyryshkin A., Shin J.N., Eissa N.T. (2020). Post-Transcriptional Regulation of Alpha One Antitrypsin by a Proteasome Inhibitor. Int. J. Mol. Sci..

[42] Rasulov C.K., Hasanov A.A., Hasanova G.J., Heydarli G.Z., Rustamov S.T. (2024). Polyphenols: General concepts and biological activity. Process. Petrochem. Oil Refin..

[43] Nian Y., Zhang Y., Ruan C., Hu B. (2022). Update of the interaction between polyphenols and amyloid fibrils. Curr. Opin. Food Sci..

[44] Eberhardt J., Santos-Martins D., Tillack A.F., Forli S. (2021). AutoDock Vina 1.2.0: New Docking Methods, Expanded Force Field, and Python Bindings. J. Chem. Inf. Model..

[45] Trott O., Olson A.J. (2010). AutoDock Vina: Improving the speed and accuracy of docking with a new scoring function, efficient optimization and multithreading. J. Comput. Chem..

[46] Krishnan B., Hedstrom L., Hebert D.N., Gierasch L.M., Gershenson A. (2017). Expression and purification of active recombinant human alpha-1 antitrypsin (AAT) from Escherichia coli. Methods Mol. Biol..

[47] Alenad A., Khan M.S., Alokail M.S., Malik A., Odeibat H., Khan S., Rehman A.A. (2025). Targeting CD33 with Hesperidin: A Natural Strategy for Acute Myeloid Leukemia Treatment. Chem. Biodivers..

[48] Rehman A.A., Zaman M., Zia M.K., Ahsan H., Khan R.H., Khan F.H. (2017). Conformational behavior of alpha-2-macroglobulin: Aggregation and inhibition induced by TFE. Int. J. Biol. Macromol..

[49] Hawe A., Sutter M., Jiskoot W. (2008). Extrinsic fluorescent dyes as tools for protein characterization. Pharm. Res..

[50] Ferguson N., Berriman J., Petrovich M., Sharpe T.D., Finch J.T., Fersht A.R. (2003). Rapid amyloid fiber formation from the fast folding WW domain FBP28. Proc. Natl. Acad. Sci. USA.

[51] Berendsen H.J.C., van der Spoel D., van Drunen R. (1995). GROMACS: A message-passing parallel molecular dynamics implementation. Comput. Phys. Commun..

[52] Panwar A., Kumar A. (2021). In-silico analysis and molecular dynamics simulations of lysozyme by GROMACS 2020. Ann. Rom. Soc. Cell Biol..

[53] Wang J., Wang W., Kollman P.A., Case D.A. (2001). Antechamber: An accessory software package for molecular mechanical calculations. J. Am. Chem. Soc..

[54] Bussi G., Donadio D., Parrinello M. (2007). Canonical sampling through velocity rescaling. J. Chem. Phys..

[55] Parrinello M., Rahman A. (1981). Polymorphic transitions in single crystals: A new molecular dynamics method. J. Appl. Phys..

[56] Kumari R., Kumar R., Lynn A. (2014). g_mmpbs-A GROMACS tool for high-throughput MM-PBSA calculations. J. Chem. Inf. Model..

[57] Louros N., Schymkowitz J., Rousseau F. (2023). Mechanisms and pathology of protein misfolding and aggregation. Nat. Rev. Mol. Cell Biol..

[58] Srisailam S., Kumar T.K.S., Srimathi T., Yu C. (2002). Influence of backbone conformation on protein aggregation. J. Am. Chem. Soc..

[59] Iram A., Naeem A. (2013). Detection and analysis of protofibrils and fibrils of hemoglobin: Implications for the pathogenesis and cure of heme loss related maladies. Arch. Biochem. Biophys..

[60] Gonçalves S., Santos N.C., Martins-Silva J., Saldanha C. (2007). Fluorescence spectroscopy evaluation of fibrinogen–β-estradiol binding. J. Photochem. Photobiol. B Biol..

[61] Calamai M., Chiti F., Dobson C.M. (2005). Amyloid fibril formation can proceed from different conformations of a partially unfolded protein. Biophys. J..

[62] Rehman M.T., Faheem M., Khan A.U. (2015). An insight into the biophysical characterization of different states of cefotaxime hydrolyzing β-lactamase 15 (CTX-M-15). J. Biomol. Struct. Dyn..

[63] Povarova O.I., Kuznetsova I.M., Turoverov K.K. (2010). Differences in the pathways of proteins unfolding induced by urea and guanidine hydrochloride: Molten globule state and aggregates. PLoS ONE.

[64] Ladiwala A.R.A., Litt J., Kane R.S., Aucoin D.S., Smith S.O., Ranjan S., Davis J., Van Nostrand W.E., Tessier P.M. (2012). Conformational differences between two amyloid β oligomers of similar size and dissimilar toxicity. J. Biol. Chem..

[65] Vondrášek J., Bendová L., Klusák V., Hobza P. (2005). Unexpectedly strong energy stabilization inside the hydrophobic core of small protein rubredoxin mediated by aromatic residues: Correlated ab initio quantum chemical calculations. J. Am. Chem. Soc..

[66] Gasymov O.K., Glasgow B.J. (2007). ANS fluorescence: Potential to augment the identification of the external binding sites of proteins. Biochim. Biophys. Acta (BBA) Proteins Proteom..

[67] Amani S., Naeem A. (2013). Detection and analysis of amorphous aggregates and fibrils of cytochrome c in the presence of phenolic acids. Int. J. Biol. Macromol..

[68] Willbold D., Strodel B., Schröder G.F., Hoyer W., Heise H. (2021). Amyloid-type protein aggregation and prion-like properties of amyloids. Chem. Rev..

[69] Sarwar T., Almatroudi A., Almatroodi S.A., Alharbi H.O.A., Rahmani A.H. (2025). In silico analysis of bioactive compounds of Nigella sativa as potential inhibitors of NS5B RdRp protein involved in the pathogenesis of hepatitis C virus. J. Biomol. Struct. Dyn..

[70] Qais F.A., Sarwar T., Ahmad I., Khan R.A., Shahzad S.A., Husain F.M. (2021). Glyburide inhibits non-enzymatic glycation of HSA: An approach for the management of AGEs associated diabetic complications. Int. J. Biol. Macromol..

[71] Fouedjou R.T., Chtita S., Bakhouch M., Belaidi S., Ouassaf M., Djoumbissie L.A., Tapondjou L.A., Abul Qais F. (2021). Cameroonian medicinal plants as potential candidates of SARS-CoV-2 inhibitors. J. Biomol. Struct. Dyn..

[72] Liao S.-Y., Mo G.-Q., Chen J.-C., Zheng K.-C. (2014). Exploration of the binding mode between (-)-Zampanolide and tubulin using docking and molecular dynamics simulation. J. Mol. Model..

[73] Siddiqui S., Ameen F., Jahan I., Nayeem S.M., Tabish M. (2019). A comprehensive spectroscopic and computational investigation on the binding of the anti-asthmatic drug triamcinolone with serum albumin. New J. Chem..

[74] Sarwar T., Obaid AAlharbi H., Alhumaydhi F.A., Khan R.M., Rahmani A.H. (2026). Computational approaches for the identification of potential HDAC2 inhibitors and histamine H3 receptor antagonists from Berberis vulgaris: A dual mechanistic approach for autism spectrum disorder treatment. Open Life Sci..

[75] Rath B., Abul Qais F., Patro R., Mohapatra S., Sharma T. (2021). Design, synthesis and molecular modeling studies of novel mesalamine linked coumarin for treatment of inflammatory bowel disease. Bioorg. Med. Chem. Lett..

[76] Siddiqui S., Ameen F., Kausar T., Nayeem S.M., Ur Rehman S., Tabish M. (2021). Biophysical insight into the binding mechanism of doxofylline to bovine serum albumin: An in vitro and in silico approach. Spectrochim. Acta Part A Mol. Biomol. Spectrosc..

[77] Luo Z., Zhao X., Zhang S. (2008). Structural dynamic of a self-assembling peptide d-EAK16 made of only d-amino acids. PLoS ONE.

[78] Vendruscolo M., Zurdo J., MacPhee C.E. (2003). Protein folding and misfolding: A paradigm of self-assembly and regulation in complex biological systems. Philos. Trans. R. Soc. Lond. Ser. A Math. Phys. Eng. Sci..

[79] Luheshi L.M., Dobson C.M. (2009). Bridging the gap: From protein misfolding to protein mis-folding diseases. FEBS Lett..

[80] Dobson C.M. (2004). Principles of protein folding: Misfolding and aggregation. Semin. Cell Dev. Biol..

[81] de la Paz Lopez M., Serrano L. (2004). Sequence determinants of amyloid fibril formation. Proc. Natl. Acad. Sci. USA.

[82] Gharibyan A.L., Zamotin V., Yanamandra K., Moskaleva O.S., Margulis B.A., Kostanyan I.A., Morozova-Roche L.A. (2007). Lysozyme amyloid oligomers and fibrils induce cellular death via different apoptotic/necrotic pathways. J. Mol. Biol..

[83] Morshedi D., Ebrahim-Habibi A., Moosavi-Movahedi A.A., Nemat-Gorgani M. (2010). Chemical modification of lysine residues in lysozyme may dramatically influence its amyloid fibrillation. Biochim. Biophys. Acta (BBA) Proteins Proteom..

[84] Sharma S., Deep S. (2024). Inhibition of fibril formation by polyphenols: Molecular mechanisms, challenges, and prospective solutions. Chem. Commun..

[85] Muzammil S., Kumar Y., Tayyab S. (1999). Molten globule-like state of human serum albumin at low pH. Eur. J. Biochem..

[86] Wu S., Liu C., Li Y., Zhang X., Han Q., Zhao H., Zhao K., Dang Y., Wang R., Song S. (2025). Inhibitory mechanisms of amentoflavone on amyloid-β peptide aggregation revealed by replica exchange molecular dynamics. Sci. Rep..

[87] Han B.H., Cofell B., Everhart E., Humpal C., Kang S.S., Lee S.K., Kim-Han J.S. (2022). Amentoflavone promotes cellular uptake and degradation of amyloid-beta in neuronal cells. Int. J. Mol. Sci..

[88] Choi E.Y., Kang S.S., Lee S.K., Han B.H. (2020). Polyphenolic biflavonoids inhibit Amyloid-beta fibrillation and disaggregate preformed Amyloid-beta fibrils. Biomol. Ther..

[89] Qiu H., Guo Z., Xu Q., Mao S., Wu W. (2021). Evaluation on absorption risks of amentoflavone after oral administration in rats. Biopharmceutics Drug Dispos..

[90] Grelle G., Otto A., Lorenz M., Frank R.F., Wanker E.E., Bieschke J. (2011). Black tea theaflavins inhibit formation of toxic amyloid-beta and alpha-synuclein fibrils. Biochemistry.

[91] Zhang G., Pan Y., Cheng H., Gong S., Chu Q., Chen P. (2022). Theaflavin: A natural candidate to restrain thrombosis. Food Funct..

[92] Qu F., Ai Z., Liu S., Zhang H., Chen Y., Wang Y., Ni D. (2021). Study on mechanism of low bioavailability of black tea theaflavins by using Caco-2 cell monolayer. Drug Deliv..

