# Supplementary material for: Biophysical and Computational Insights into Alpha-1 Antitrypsin Aggregation and Its Inhibition by Natural Polyphenols
Source: Biomedicines. 2026 Jun 9;14(6):1310. doi: 10.3390/biomedicines14061310 (PMC13297509; doi:10.3390/biomedicines14061310)
Supplement: Supplementary file 1 [file biomedicines-14-01310-s001.zip › biomedicines-4280937-supplementary.pdf]

Supplementary Materials

Biophysical and Computational Insights into Alpha-1 Antitrypsin Aggregation and Its Inhibition by Natural Polyphenols

Tarique Sarwar [1], Ahmed Abdur Rehman [2], Hussain Arif [2], Wanian M. Alwanian [1], Hajed Obaid A. Alharbi [1] and Arshad Husain Rahmani [1,*]

[1] Department of Medical Laboratories, College of Applied Medical Sciences, Qassim University, Buraydah 51452, Saudi Arabia; t.sarwar@qu.edu.sa (T.S.); w.alwanian@qu.edu.sa (W.M.A.); hajed.alharbi@qu.edu.sa (H.O.A.A.)

[2] Department of Biochemistry, Faculty of Life Sciences, Aligarh Muslim University, Aligarh 202002, India; ahmedarehman@gmail.com (A.A.R.); arifkap@gmail.com (H.A.)

* Correspondence: ah.rahmani@qu.edu.sa

Figure S1: RMSF of each atom of ligands AMF and TF during 100 ns MD simulation.

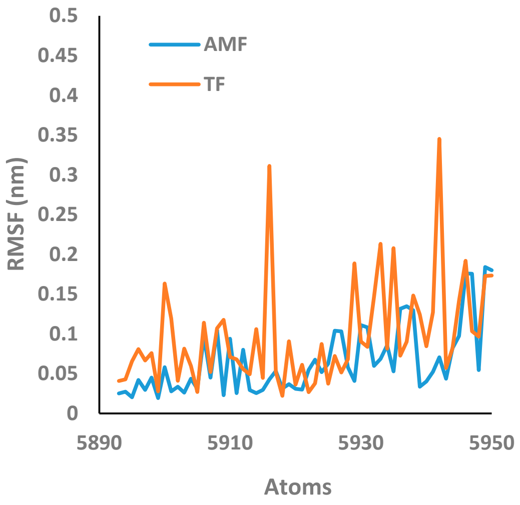

Table S1: Molecular docking-based screening data and affinity values (binding free energies in kcal/mol) of alpha-1 antitrypsin (A1AT) with phytocompounds.

S. No.	Name of the Ligand	PubChem CID	Binding Free Energy (kcal/mol)
1.	Amentoflavone	5281600	-8.6
2.	Theaflavin	135403798	-8.2
3.	Poncirin	442456	-8.1
4.	Silybin	31553	-7.8
5.	Quercitrin	5280459	-7.8
6.	Azalein	5321320	-7.6
7.	Malvin	441765	-7.6
8.	Antirrhinin	441674	-7.5
9.	Hypolaetin	5281648	-7.4
10.	Epicatechin Gallate	107905	-7.4
11.	Luteolin	5280445	-7.3
12.	Epicatechin	72276	-7.3
13.	Spiraeosid	5320844	-7.3
14.	Apigenin	5280443	-7.2
15.	Naringenin	932	-7.2
16.	Daidzin	107971	-7.2
17.	Sakuranin	73607	-7.2
18.	Pulchellidin	14496545	-7.1
19.	Amurensin	5318156	-7.1
20.	Hidrosmin	3087722	-7.1
21.	Pinocembrin	68071	-7.0
22.	Diosmetin	5281612	-7.0
23.	Sterubin	1268276	-7.0
24.	Pelargonidin	440832	-6.9
25.	Dactylifric acid	139031052	-6.9
26.	Rosmarinic acid	5281792	-6.9
27.	Gallocatechol	1249	-6.8
28.	Puerarin	5281807	-6.8
29.	Chlorogenic acid	1794427	-6.8
30.	Primulin	3568969	-6.8
31.	Ombuin	5320287	-6.7
32.	Annulatin	44259709	-6.7
33.	Delphinidin	128853	-6.7
34.	Petunidin	441774	-6.6
35.	Dactylifric acid	139031052	-6.6
36.	Chicoric acid	5281764	-6.5
37.	Rosinidin	441777	-6.4
38.	Syringetin	5281953	-6.4
39.	Caftaric acid	6440397	-6.4
40.	Sinensetin	145659	-6.3
41.	Malvidin	159287	-6.3

**42.**	Arbutin	440936	-6.1
**43.**	Umbellic acid	446611	-5.8
**44.**	Tangeritin	68077	-5.8
**45.**	Xanthoplanine	14262868	-5.7
**46.**	Caffeic acid	689043	-5.7
**47.**	Vanillic acid	8468	-5.6
**48.**	Salicylic acid	338	-5.5
**49.**	Gallic acid	370	-5.5
**50.**	Ferulic acid	445858	-5.4

Table S2: The number of residues falling into various categories across all energy minima structures for A1AT, A1AT-AMF, and A1AT-TF complex.

	A1AT		A1AT-AMF		A1AT-TF	
**Categories**	**Number of Residues**	**Percentage**	**Number of Residues**	**Percentage**	**Number of Residues**	**Percentage**
**Residues in most favoured regions [A,B,L]**	274	82.5%	285	85.8%	288	86.7%
**Residues in additional allowed regions [a,b,l,p]**	57	17.2%	46	13.9%	42	12.7%
**Residues in generously allowed regions [~a,~b,~l,~p]**	1	0.3%	1	0.3%	1	0.3%
**Residues in disallowed regions**	0	0.0%	0	0.0%	1	0.3%
**Number of non-glycine and non-proline residues**	332	100%	332	100%	332	100%
